# Loss of FGF-Dependent Mesoderm Identity and Rise of Endogenous Retinoid Signalling Determine Cessation of Body Axis Elongation

**DOI:** 10.1371/journal.pbio.1001415

**Published:** 2012-10-30

**Authors:** Isabel Olivera-Martinez, Hidekiyo Harada, Pamela A. Halley, Kate G. Storey

**Affiliations:** 1 Division of Cell and Developmental Biology, College of Life Sciences, University of Dundee, Dundee, United Kingdom; 2 Department of Molecular Neurobiology, Graduate School of Life Sciences and Institute of Development, Aging and Cancer, Tohoku University, Sendai, Japan; The Wellcome Trust Sanger Institute, United Kingdom

## Abstract

The endogenous mechanism that determines vertebrate body length is unknown but must involve loss of chordo-neural-hinge (CNH)/axial stem cells and mesoderm progenitors in the tailbud. In early embryos, Fibroblast growth factor (FGF) maintains a cell pool that progressively generates the body and differentiation onset is driven by retinoid repression of FGF signalling. This raises the possibility that FGF maintains key tailbud cell populations and that rising retinoid activity underlies cessation of body axis elongation. Here we show that sudden loss of the mesodermal gene (Brachyury) from CNH and the mesoderm progenitor domain correlates with FGF signalling decline in the late chick tailbud. This is accompanied by expansion of neural gene expression and a similar change in cell fate markers is apparent in the human tailbud. Fate mapping of chick tailbud further revealed that spread of neural gene expression results from continued ingression of CNH-derived cells into the position of the mesoderm progenitor domain. Using gain and loss of function approaches in vitro and in vivo, we then show that attenuation of FGF/Erk signalling mediates this loss of Brachyury upstream of Wnt signalling, while high-level FGF maintains Brachyury and can induce ectopic CNH-like cell foci. We further demonstrate a rise in endogenous retinoid signalling in the tailbud and show that here FGF no longer opposes retinoid synthesis and activity. Furthermore, reduction of retinoid signalling at late stages elevated FGF activity and ectopically maintained mesodermal gene expression, implicating endogenous retinoid signalling in loss of mesoderm identity. Finally, axis termination is concluded by local cell death, which is reduced by blocking retinoid signalling, but involves an FGFR-independent mechanism. We propose that cessation of body elongation involves loss of FGF-dependent mesoderm identity in late stage tailbud and provide evidence that rising endogenous retinoid activity mediates this step and ultimately promotes cell death in chick tailbud.

## Abstract 2

By analyzing cellular and molecular changes in key cell populations in the tailbud during embryogenesis, this work uncovers critical signaling events that determine vertebrate body length.

## Author Summary

The mechanism that determines body length is unknown but likely operates at the elongating tail end of vertebrate embryos. In the early embryo, fibroblast growth factor (FGF) signalling maintains a proliferative pool of cells in the tailbud that progressively generates the body. It also protects these cells from the differentiating influence of retinoic acid, which is produced by the maturing mesoderm tissues of the extending body. We show here, in the chick embryo, that the “endgame”—that is, the termination of body axis elongation—comes when the mesodermal gene brachyury is suddenly lost from axial stem cell population and presumptive mesoderm cells in the tailbud late in development. Using gain- and loss-of-function approaches, we demonstrate that this step is mediated by loss of FGF signalling. We present evidence that this is due to rising retinoid signalling in the tailbud and that FGF signalling in the tailbud no longer opposes retinoid synthesis and activity. Finally, we reveal that these events are followed by local cell death in the tailbud, which can be reduced by the attenuation of retinoid signalling but involves a mechanism that is independent of FGF signalling via its usual receptor. We propose that cessation of body elongation involves loss of FGF-dependent mesoderm identity in the late tailbud and that this is mediated by rising endogenous retinoid activity, which ultimately promotes cell death in the chick tailbud.

## Introduction

**Figure 1:**
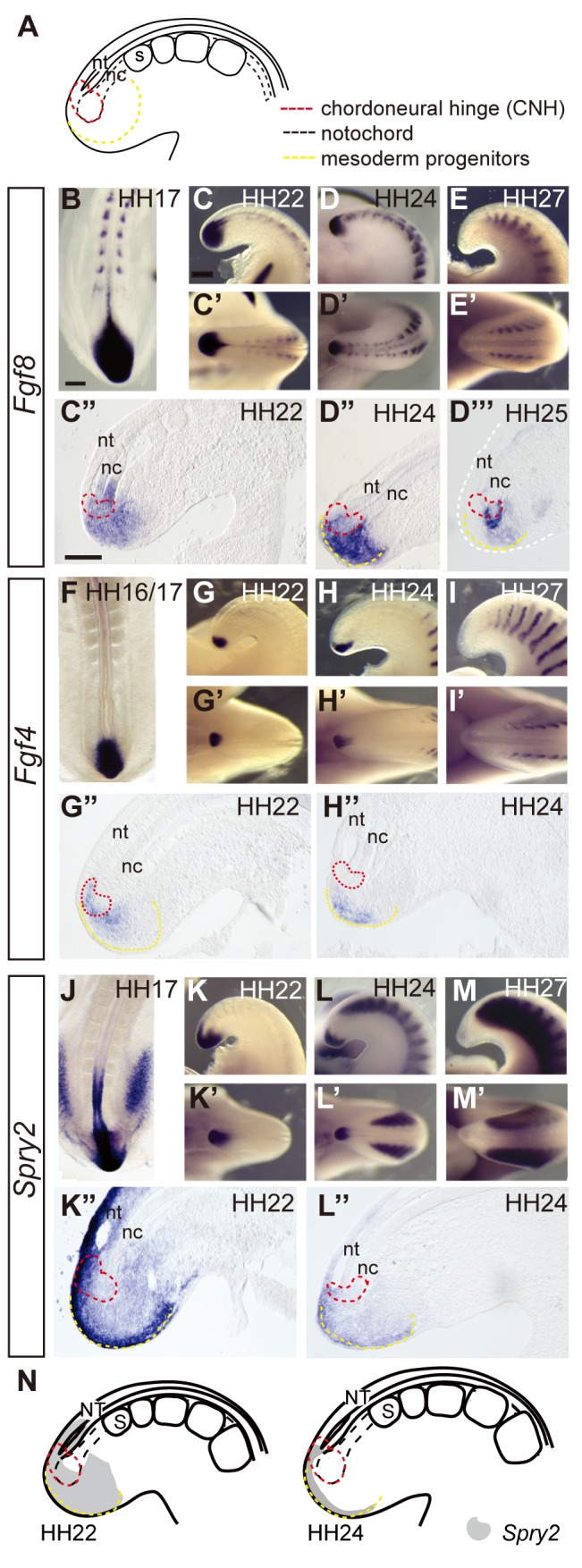
Key tailbud cell populations and changing FGF pathway ligand expression and activity in the maturing tailbud. (A) Schematic of key tailbud tissues; chordoneural hinge (red dashed line) consists of caudal-most ventral neural tissue and distal end of notochord (black dashed line within red dashed line) and presomitic mesoderm progenitors (yellow dashed line). These cell populations are defined by position, morphology, and their fates, following mapping studies [5] and data below. In situ hybridisation during body axis elongation *Fgf8* (B–E′), *Fgf4* (F–I′), and *Spry2* (J–N). In all figures, top rows are lateral views, bottom rows dorsal views, and sections are sagittal unless indicated otherwise. nt, neural tube; nc, notochord; s, somite. Scale bars in all figures are 100 µm.

Cells located in the tailbud of the vertebrate embryo generate the body progressively. These cell populations include axial stem cells in the chordoneural hinge (CNH, classically defined as caudal-most ventral neural tissue and distal notochord) that contribute to notochord, somites, and ventral neural tube in a self-renewing manner [1]–[3] and more caudally located somitic mesoderm progenitors, which have a limited self-renewing ability (Figure 1A) [4],[5]. Extrinsic signals, including Wnt and FGF, are required for continued body axis elongation in the early embryo (reviewed in [2]), and this process relies on the regulated differentiation of newly generated cells as they exit the tail end. At a specific point, however, body axis elongation ceases and this must involve the regulated differentiation and/or loss of axial stem and mesoderm progenitor cells.

Changes in a number of signalling pathways can induce axial truncations in the early embryo, although how they act on specific cell populations in the later forming tailbud has not been explored. Exposure to exogenous retinoic acid (RA) can arrest body elongation [6],[7] by inhibiting expression of *Wnt3a* [7]–[9]. A critical level of bone morphogenetic protein signalling is also required for normal axis elongation; loss of the bone morphogenetic protein antagonist Noggin or Noggin overexpression both generate a truncation phenotype, reviewed in [2]. Loss of FGF signalling also leads to body axis truncation at early, pre-tailbud stages, reviewed in [2]. Importantly, Wnt3a is required for maintenance of *Fgf8* in the caudal regions [10], and this regulatory relationship provides a link from Wnt signalling to mechanisms that protect tail-end cells from endogenous RA, all of which in higher vertebrates are, so far, downstream consequences of FGF signalling. FGF signals maintain expression of *Cyp26A* (which catabolises RA) in the tail end, repress expression of the retinoic acid receptor *RARß* in the neuroepithelium, and critically inhibit expression of the retinoid synthesising enzyme retinaldehyde dehydrogenase 2 (*Raldh2)* in the forming somites [11]–[13]. At early stages while FGF signalling antagonises the RA pathway, RA in turn drives differentiation in neural and mesodermal tissues by inhibiting expression of *Fgf8* [11],[14]. However, while cells generated at the tail end continue to experience sequentially FGF and then retinoid signalling as they leave this region, it is not known whether this mutual antagonism between these signalling pathways is maintained throughout development. Indeed, it is clear that as the body axis elongates in chick and mouse, the presomitic mesoderm initially gets longer, separating FGF and RA signalling centres, but then shortens following tailbud formation. The tailbud forms after about half the body axis has been generated, from the remnants of the primitive streak and adjacent epiblast or stem zone, and in the chick generates body axis tissues from the level of somites 27–26 to ∼52 and in mouse from somite 35 to ∼65 ([15],[16]; reviewed in [2]). So although established in early embryo, it has yet to be determined whether FGF still represses *Raldh2* at tailbud stages or if RA can still inhibit expression of Fgf ligands and, as somite formation nears the tailbud, finally overwhelm FGF signalling to arrest axis elongation. A further indication that this may be the case is the recent observation that *Raldh2* appears in a new domain within the tailbud itself [17].

Mutation of several transcription factors also truncates the body axis. These include Cdx and Hox genes that are part of a positive regulatory loop with FGF and Wnt signalling pathways [18]–[22]. The T-box factor, Brachyury (*Bra*) or T also controls body axis elongation as indicated by the T-mouse mutant phenotype [23],[24]. In zebrafish, retinoic acid represses Bra/Ntl, which normally protects mesoderm progenitors in the tailbud from retinoid signalling by inducing expression of *Cyp26a* [25]. However, it is not clear that these regulatory relationships are conserved in higher vertebrates, as caudal *Cyp26a* expression is dependent on FGF signalling in chick and mouse embryos [13],[26], while in zebrafish neither *Cyp26a* nor *Bra/Ntl* require FGF [25]. It is also worth noting that different enhancers control the expression of *Bra* in distinct domains in the notochord and paraxial mesoderm [27],[28] and that little is known about how *Bra* expression is regulated in distinct cell populations within the mature tailbud nor how these might be influenced by retinoid signalling.

Exogenous RA and loss of *Wnt3a* can induce programmed cell death in the tailbud, however such signalling events do not always elicit apoptosis when the body axis is truncated [18],[29]. It is not known whether endogenous RA signalling acts in the tailbud by promoting programmed cell death nor if this takes place in specific cell populations at key times within the maturing tailbud. Intriguingly, an imbalance of retinoid, Wnt3a, or FGF signalling can also alter the neural versus mesodermal cell fate decision in caudal regions of the early embryo [8],[9],[29]–[34]. Rising endogenous RA activity in the late tailbud might therefore act by altering signals that maintain the axial stem cell niche and/or mesoderm progenitors, resulting in loss of multipotency and diversion towards neural fate.

Although many gene regulatory relationships are conserved between vertebrate embryos, the development of caudal-most structures seems to differ; in humans, this appears to be closest to that observed in the chick embryo: both undergo secondary neurulation and caudal regression and lack the extended tail characteristic of the mouse. Indeed, recent work has shown that the chick but not the mouse tailbud is an endogenous source of RA [17],[35], raising the possibility that distinct mechanisms may operate in different species. Here we address the mechanism of body axis termination in the chick by tracking FGF and retinoid signalling dynamics in specific tailbud cell populations and correlate this with cell movements and cell specification changes as elongation ceases. Using in vivo and in vitro approaches we demonstrate that continued generation of mesoderm along the body axis, as indicated by Brachyury expression, depends on FGF signalling and show that it is attenuated by rising retinoid signalling, which also eventually promotes cell death.

## Results

### Fgf Signalling Persists in the Tailbud and Declines Prior to Axis Elongation Arrest

The expression patterns of key FGF pathway genes were examined in detail in distinct tailbud cell populations (see Figure 1A) at stages of tailbud formation and maturation in the chick (Hamburger and Hamilton [HH] stage16/17 to HH27) [36]. The FGF ligands *Fgf8* and *Fgf4* were strongly expressed in the tailbud but declined in the last 2 days of axis elongation from HH22 (day 4) to HH26/7 (day 6) (Figure 1B–E′ and Figure 1F–1I′) (with the segmentation of the paraxial mesoderm ceasing at HH24 [15],[16]). Initially, *Fgf8* is expressed in the caudal-most neural tube and distal notochord, which together form the chordoneural hinge (CNH) and in the surrounding mesoderm progenitors (Figure 1C″) and is downregulated by HH24/25 in mesoderm progenitors, neural CNH, and most notochord CNH (Figure 1D″, 1D′″). *Fgf4* is restricted to mesoderm progenitors at all stages in the tailbud (Figure 1G–1H″) and is similarly lost as this tissue matures (Figure 1F–1H″). The expression pattern of the FGF/Erk feedback antagonist *Sprouty2* (*Spry2*) serves as a reporter for FGF activity [37] and is detected in caudal neural tube, mesoderm progenitors, and CNH at all stages to HH22 (Figure 1J–K″) but is lost from the neural CNH and reduced in the mesoderm progenitors by HH24 (e.g., compare Figure 1K–1K″ to 1L–1L″). Transcripts for Fgf pathway ligands and *Spry2* are then lost completely from the tailbud by HH26/27 (Figure 1E, 1E′, 1I, 1I′ and 1M, 1M′). These expression patterns indicate a rapid decline in FGF signalling in neural CNH and mesoderm progenitors in the mature HH24 tailbud (summarised by *Spry2* expression in Figure 1N).

### Loss of Mesodermal and Spread of Neural Gene Expression in the Late Tailbud

**Figure 2:**
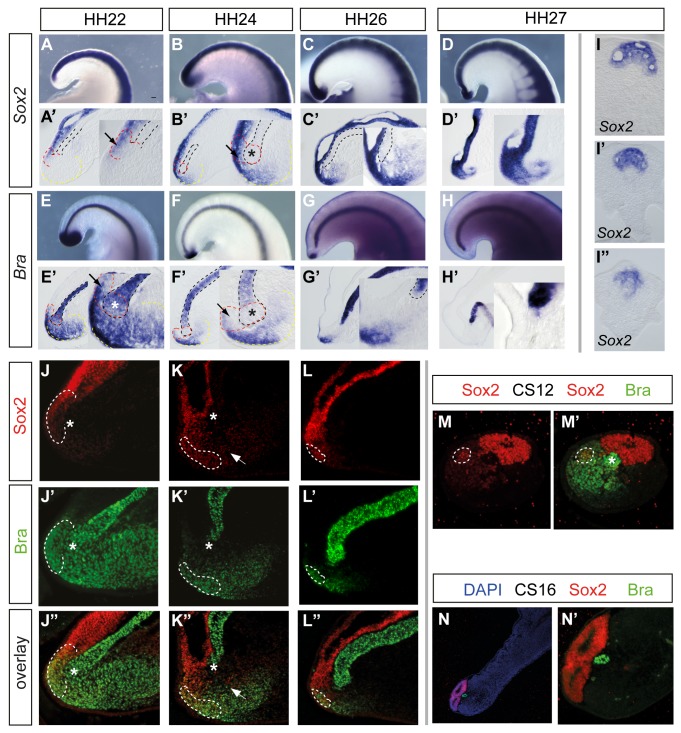
Dynamics of Sox2 and Bra expression in chick and human tailbuds. (A–D) Neural progenitor marker *Sox2* mRNA expands into the mesoderm progenitor domain from HH24 (arrows in A′ and B′) analysed in HH22–27 stages in wholemount (A–D) and in medial saggital sections (SS) (A′–D′) at low and high magnification. The mesoderm progenitor marker *Brachyury* (*Bra*) is expressed in notochord, CNH, and caudal presomitic mesoderm at HH22. Expression is lost from the distal notochord/CNH (* in E′ and F′) and from neural CNH and mesoderm progenitors (arrows in E′ and F′) from HH24–27 analysed in wholemount (E–H) and in medial saggital sections (E′–H′). Transverse sections from rostral to caudal through HH26–27 tailbud showing termination in *Sox2*-expressing tissue with multiple lumens (I–I″). Immunocytochemistry for Sox2 and Bra in chick tailbuds at HH22 (J–J″). Overlap of Sox2 and Bra is confined to the neural-CNH (white dashed line), and Bra domain is continuous between notochord and CNH and mesoderm progenitors (indicated by * marking distal notochord). At HH24 (K–K″), Bra is lost from distal notochord (*) and surrounding medial mesoderm progenitors, as well as the neural CNH (which is reduced in size; white dashed line), while Sox2-expressing cells are now located between the notochord and CNH and in the “mesoderm progenitor” domain at HH26. (L–L″) Bra is detected at the caudo-lateral edge of the mesoderm progenitor domain, and the Sox2/Bra-expressing CNH is reduced still further (white dashed line). (M) Human tailbud showing Sox2 expressing cells in the neural tube and in the mesoderm progenitor domain (white dashed line) at CS12. (M′) Expression of Sox2 and Bra in the CS12 tailbud; the highest levels of Bra are detected in the distal notochord (*). (N) Human tailbud showing terminal neural structures and distal notochord at CS16. (N′) High-magnification view of tailbud shown in (N); Bra-expressing mesodermal progenitor cell population is no longer present at the tail tip, which terminates in a multi-lumen neural tube. Colour code for dashed lines as in Figure 1A.

In the early embryo, FGF activity is required to promote mesodermal over neural cell fate, and so declining FGF signalling might cause body axis elongation to cease if normal cell fate specification is lost in the tailbud. To examine whether cell fates change in specific cell populations in the maturing tailbud, we analysed expression of two key marker genes in detail. Strikingly, the neural progenitor marker *Sox2* was found to expand into the positionally defined mesodermal progenitor domain between HH22–24, separating the distal swelling of the notochord from the mesoderm progenitors (Figure 2A–2C′) (*Sox2* is also detected in the remnant of the tail gut at HH20 (Figure S1), but is lost as this cell population degenerates by HH22 [38]). Concomitant with expansion of the *Sox2* domain, transcripts of the early mesoderm marker *Bra* were lost from the position of the medial mesoderm progenitors, which surround the notochord tip (arrows in Figure 2B′ and 2F′). In addition, *Bra* was downregulated in the CNH (both the distal notochord swelling and caudal-most ventral neural tube) (Figure 2F–2H′). This dramatic local change in gene expression correlates with the loss of the morphologically defined contiguous notochord/CNH (asterisks in Figure 2E′, 2F′). These observations suggest that the axial stem cell population of the CNH and the “mesodermal progenitors” in the tailbud lose their mesoderm potential coincident with cessation of segmentation at HH24. At later stages, the broadened *Sox2*-expressing domain then differentiated into a multi-lumen neural tube (Figure 2I–2I″).

This critical change in gene expression was further assessed at the protein level by analysis of double immunocytochemistry for Bra and Sox2 at HH22, HH24, and HH26 (Figure 2J–L″). This revealed a sharp loss of Bra protein in the distal notochord tip and surrounding cells in the position of mesoderm progenitors (asterisks in Figure 2J′, 2K′) and a reduction in Bra in the neural CNH (defined by Sox2/Bra co-expressing cells continuous with the caudal-most neural tube), which progressively diminishes from HH24 (Figure 2J″–L″). In addition, in the positionally defined mesoderm progenitor domain, Sox2 positive cells (white arrows in Figure K, K″) were also apparent as well as some Sox2/Bra co-expressing cells (which here may reflect a transitional cell state as Bra is lost in this cell population).

Importantly, analysis of these proteins in serial sections of the human tailbud at Carnegie Stage (CS) 12 (26–30 days, 21–29 somites, equivalent to early tailbud HH18 in terms of somite number) (*n* = 2) and at CS16 (37–42 days, 38–39 somites; corresponds to the end of axis elongation [39] equivalent to HH26/27) (*n* = 1) revealed a similar loss of CNH (Sox2/Bra cell population) and of Bra expression as this tailbud matures (Figure 2M–N′). At CS12 Sox2 was detected at high levels in neural tube, but also in an adjacent discrete cell group that co-expresses Bra, which may be equivalent to the CNH (Figure 2M, 2M′). Bra was also detected at high levels in notochord at CS12 (Figure 4M′). Analysis of serial sections through the later CS16 human tailbud revealed that by the end of somitogenesis this was capped at its terminal end by Sox2 expressing neuroepithelium and Bra was now confined to the notochord (Figure 2N, 2N′). These observations indicate that arrest of body axis elongation in chick and human is characterised by loss of Bra expression in the tailbud, leaving terminal cells with a neural character.

### Fate Mapping the Late Tailbud Demonstrates Continued Cell Ingression from the CNH

**Figure 3:**
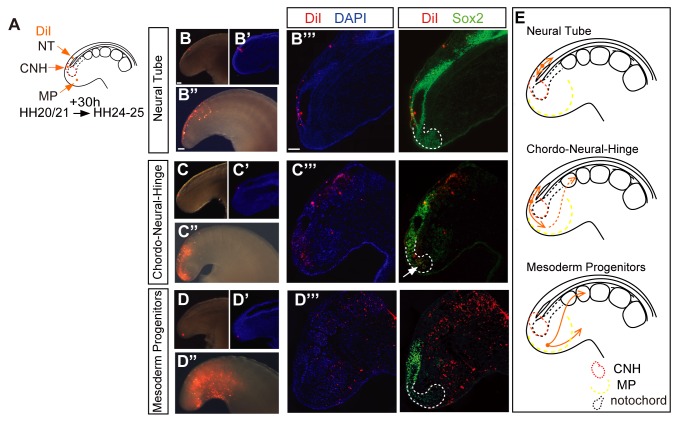
Fate mapping the late tailbud reveals continued cell ingression. Schematic of DiI labelling experiment for distinct cell populations in HH20/21 tailbud (NT, neural tube; CNH, chordo-neural-hinge; MP mesoderm progenitors) (A); DiI labelling of NT before incubation (B), fixed and analysed in sections (*n* = 5/5 confined to NT) (B′); after incubation, DiI is restricted to Sox2 positive NT (B′″); DiI labelling of CNH before incubation (C), fixed and analysed in sections (*n* = 8/11 confined to CNH) (C′); after incubation, DiI is found in NT, MPs, and their derivatives, including Sox2 positive cells in the MP domain (C′″); DiI labelling of MPs before incubation (D), fixed and analysed in sections (*n* = 6/6 confined to MPs) (D′); after incubation, DiI is restricted to MPs, presomitic mesoderm, somites, and lateral mesoderm and rarely labels Sox2 positive cells in the MP domain (D′″). White arrow, Sox2-expressing/DiI labelled cells in MP domain; white dashed line, region of Sox2 positive cells in the position of the MP domain. (E) Summary of cell movements following DiI labelling (orange) in NT, CNH, and MP domain.

To understand the contribution that cell movement might make to these changing patterns of gene expression, a series of fate mapping studies were carried out in the tailbud in ovo (Figure 3A and see Materials and Methods). DiI was used to label focal groups of ∼20–40 cells in the caudal-most neural tube, the “neural CNH,” or the mesoderm progenitor domain at HH20 (local labelling was confirmed by observation in the whole embryo, and to ensure accurate labelling, a subset of embryos were fixed immediately and sectioned to define position of DiI labelled cells; Figure 3B–3D, Table 1). After 30 h incubation (to HH24/25), neural tube cells were found to contribute only to neural tissue (Figure 3B′–3B′″, Table 1), while cells in the CNH contribute to neural tissue and the positionally defined mesoderm progenitor domain, presomitic mesoderm, and somites (Figure 3C–3C′″, Table 1). This suggests that at least some of the Sox2 positive cells that appear in this “mesoderm progenitor” territory at HH24 are derived from the CNH (Figure 3C′″). We also wished to ascertain whether cells present in the *Bra* positive mesoderm progenitor domain at HH20 (prior to the appearance of Sox2 here at HH24) later come to express Sox2. Mesoderm progenitors labelled at HH20 were therefore assessed for Sox2 expression at HH24. However, the majority of DiI-labelled cells left the mesoderm progenitor domain and contributed to pre-somitic, somitic, and/or more lateral mesoderm, and few remaining cells were in the new Sox2 expressing domain (Figure 3D′″, Table 1). These data (summarized in Figure 3E) therefore suggest that Sox2 expansion into the tailbud core is due to maintenance of this gene and loss of *Bra* in cells that have recently ingressed from the CNH.

**Table 1:** Summary data for fate map of the late tailbud.

Tissue Labelled	*n*	NT	CNH	MP	Pre-Somitic Mesoderm	Somite	Lateral Mesoderm
Neural tube	8	8	1	0	0	0	0
Chordo-neural hinge	12	12	12	11	7	6	7
Mesoderm progenitors	11	0	0	9	9	9	11

Summary of contributions of cell groups DiI labelled in HH20 neural tube (NT), chordoneural hinge (CNH), or mesoderm progenitors (MP) to tissues in and derived from the tailbud observed at HH24. *n*, number of embryos labelled at HH20 for each tissue.

### FGF Signalling Is Required to Maintain Mesodermal Gene Expression in the Tailbud

**Figure 4:**
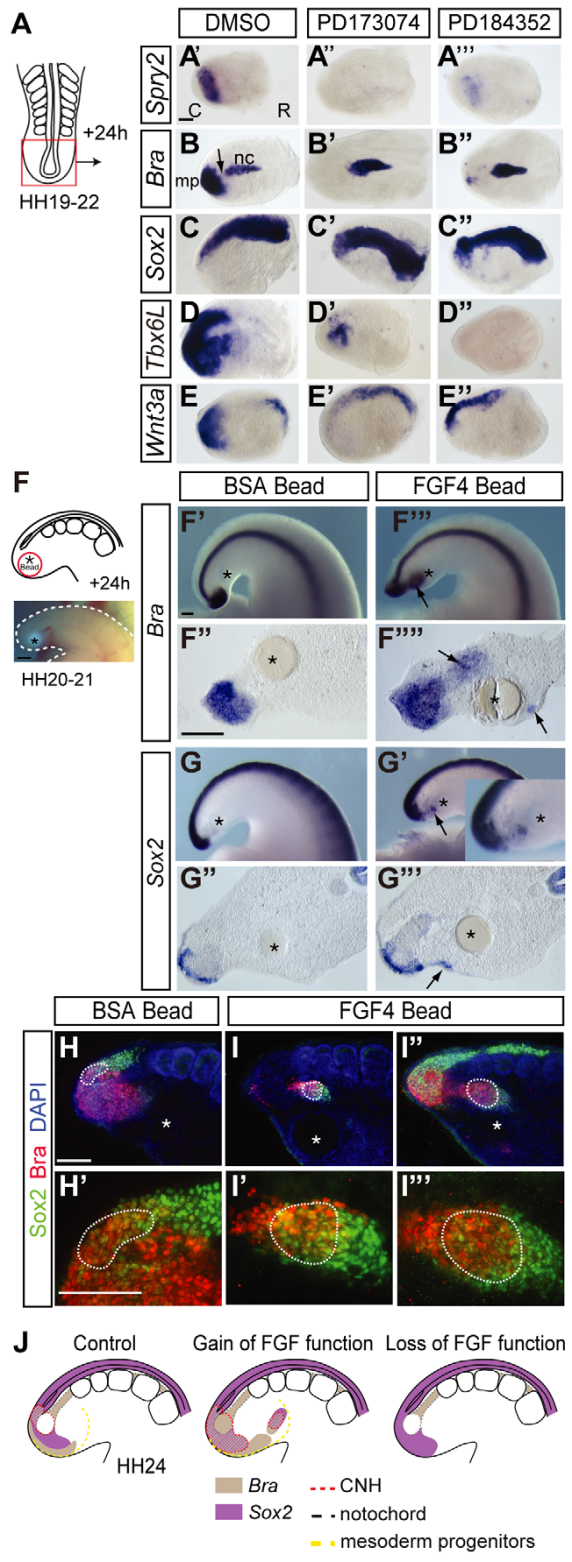
FGF signalling regulates cell fate assignment in the tailbud. Schematic of whole tailbud explant assay (A); *Spry2* expression in tailbud explants following exposure to vehicle control DMSO (A′), FGFR inhibitor PD173074 (A″), or MEK antagonist PD184352 (A′″); *Bra* expression in tailbud explants following exposure to DMSO (B), PD173074 (B′), or PD184352 (B″). Arrow indicates normal down regulation of *Bra* in CNH in DMSO control; nc, notochord; mp, mesoderm progenitors. *Sox2* expression in tailbud explants following exposure to DMSO (C), PD173074 (C′), or PD184352 (C″); *Tbx6L* expression in tailbud explants following exposure to DMSO (D), PD173074 (D′), or PD184352 (D″); *Wnt3a* expression in tailbud explants following exposure to DMSO (E), PD173074 (E′), or PD184352 (E″). In all tailbud explant experiments, treatment groups were processed and scored alongside control DMSO tailbuds; for simplicity of presentation, one representative DMSO explant is shown here for each marker gene. Each tailbud was scored for gene expression in caudal, middle, and rostral thirds. Schematic of bead implantation experiment and image of tailbud with implanted bead prior to incubation (F); vehicle control BSA-only soaked bead does not induce *Bra* (F′) and sagittal section (F″); FGF4 delivering bead eliciting ectopic *Bra* expression (F′″) and sagittal section (F″″); BSA beads do not elicit ectopic *Sox2* (G), but FGF4 beads can induce a small patch of *Sox2* (G′); BSA beads do not elicit ectopic Sox2/Bra cell foci (H–H′), but these are detected around FGF4 beads (I–I′″); note polarised organisation with co-expressing cells flanked by Sox2 and Bra only expressing cells (I′ and I′″) (arrows in F′, F′″, G′, G′″, ectopic expression domains; asterisk, beads; white dashed line, H′ endogenous CNH or ectopic CNH-like cell group I′,I′″). (J) Summary diagrams depicting combined results of FGF gain- and loss-of-function experiments. Gain of FGF function depicts effects of local exposure to FGF delivered by an implanted bead.

The loss of Bra and expansion of the Sox2 expression domain into the tailbud at HH24 correlate well with the decrease in FGF signalling in the mesoderm progenitor domain and neural CNH (summarized in Figure 1N and compare Figure 1K″ and 1L″ with Figure 2E′ and 2F′). To test whether decline in FGF signalling underlies this striking change, FGF signalling was blocked in HH19–22 tailbuds using the Fgf receptor inhibitor PD173074 or the Mitogen-activated protein kinase kinase (MEK) antagonist PD184352, which blocks downstream Erk1/2 activity. Control tailbuds exposed to DMSO alone expressed *Spry2* in the mesoderm progenitor domain (Figure 4A) and *Bra* in the mesoderm progenitors and notochord (while *Bra* expression in intervening CNH/medial mesoderm progenitors was downregulated as normal) (arrow in Figure 4B), *Sox2* expression extended to the tailbud tip (Figure 4C), and *Tbx6L* was expressed in mesoderm progenitors and extended into caudal presomitic mesoderm (Figure 4D); all gene expression patterns typical of the normal HH23–24 tailbud. Exposure to PD173074 or PD184352 inhibited expression of *Spry2* (DMSO *n* = 1/16, PD173074 *n* = 14/16; DMSO *n* = 1/4, PD184352 *n* = 3/4) (Figure 4A–4A′″) and repressed all *Bra* expression except in the proximal notochord (*Bra*, DMSO *n* = 0/10, PD173074 *n* = 10/10; DMSO *n* = 0/4, PD184352 *n* = 6/7; Figure 4B–B″), while *Sox2* transcripts were more widely detected caudally in comparison with controls (*Sox2*, DMSO *n* = 1/6, PD173074 *n* = 4/7; DMSO *n* = 0/3, PD184352 *n* = 3/5; Figure 4C–4C″). A recent study has shown that the further T-box gene *Tbx6* is required to downregulate *Sox2* expression in the early mouse embryo [40]. The homologous gene *Tbx6L* is downregulated as the chick tailbud matures [17], and so its loss provides a potential mechanism to explain the upregulation of *Sox2* in the mesoderm progenitor cell population. Importantly, blocking FGFR- or MEK-mediated signalling also inhibited *Tbx6L* (*Tbx6L*, DMSO *n* = 0/7, PD173074 *n* = 7/7; DMSO *n* = 0/10, PD184352 *n* = 11/11; Figure 4D–4D″), demonstrating that maintenance of this further mesoderm identity gene is dependent on FGF/Erk activity. As *Bra* is a known direct target of Wnt signalling, we also assessed the effects of FGFR and MEK inhibition on expression of the key ligand *Wnt3a*. This gene is expressed in tailbud mesoderm and dorsal neural tube, and its expression was repressed by both FGFR or MEK antagonists specifically in the tailbud mesoderm (DMSO *n* = 0/3; PD173074 *n* = 8/8; DMSO *n* = 0/6, PD184352 *n* = 10/10; Figure 4E–4E″).

Finally, grafting an FGF4 delivering bead into the HH20–22 tailbud induced ectopic expression of *Bra* (BSA bead control *n* = 0/7; FGF4 beads *n* = 4/6 embryos; Figure 4F–4F″″). FGF4 beads were also found to elicit an ectopic patch(es) of *Sox2* expression in cells close to the bead (BSA *n* = 0/6; FGF4 *n* = 5/7; Figure 4G–4G″). This initially surprising result may reflect the regulation of *Sox2* by distinct enhancer elements active around the organiser region (N1) and in the developing spinal cord (N4) [41]; expansion of *Sox2* expression on FGF inhibition (results above) may be indicative of spinal cord differentiation via N4 activity, while induction of *Sox2* by high-level FGF may reflect the N1 element, which is FGF/Wnt responsive [42] and might be indicative of formation of an ectopic CNH.

To address if a CNH-like state is induced, we determined whether FGF-induced ectopic Sox2 positive cells co-express Bra, as observed in the endogenous CNH. In most cases, ectopic co-expression was detected, suggesting that these cells have CNH character (BSA *n* = 0/4; Figure 4H–4H′; FGF4 *n* = 4/6; Figure 4I–4I′). Some of the ectopic Sox2/Bra cells formed small groups, but we also detected strong Sox2 expression in Bra positive cells in mesoderm progenitor domain/presomitic mesoderm in tails with FGF beads (Figure 4I–4I′″). In 3/6 embryos, ectopic Sox2/Bra foci exhibited a polarised configuration, being flanked at either end by Sox2 or Bra only expressing cells, reminiscent of tissue organisation around the endogenous CNH (Figure 4I′–4I′″). Together these findings (summarised in Figure 4J) therefore suggest that maintenance of mesoderm progenitors and the CNH depends on FGF/Erk signalling and that reduction of such signalling leads to neural differentiation in the tailbud, while high-level FGF activity can promote a CNH-like cell state.

### Key Components of the Retinoid Pathway Are Detected in the Late Stage Tailbud

**Figure 5:**
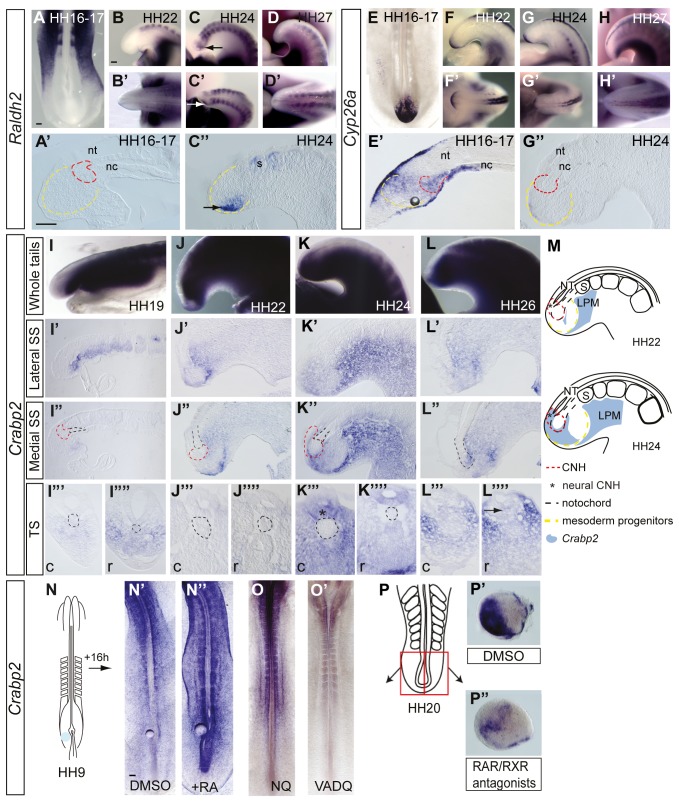
Expression of retinoid pathway genes in the maturing tailbud. *Raldh2* expression in the maturing tailbud (A–D′). Arrows indicate new tailbud *Raldh2* domains in the caudo-lateral mesoderm progenitors (C–C″). *Cyp26a* expression is downregulated in the early tailbud and is absent by HH24 (E–H′). Gradual expansion of *Crabp2* transcription into chick tailbud from HH19 to HH26 in whole-mount (I–L), analysed in lateral (I′–L′) and medial (I″–L″) sagittal sections (SS) and in transverse sections (TS) of rostral (I′″–L′″) and caudal (I″″–L″″) tailbud. Black dashed lines indicate distal tip of notochord in (SS) and notochord in TS; red dashed lines indicate CNH at stages where this can be defined (HH19, 22, 24). Asterisk in (K′″) indicates neural CNH. Summary of changes in *Crabp2* expression between HH22 and HH24, indicating increasing RA activity in mesoderm progenitors and neural portion of the CNH and at edges of notochord CNH as body axis elongation ceases at HH24 (M). Bead grafting schematic (N). Expression of *Crabp2* in chicken embryos grafted with DMSO bead (N′) or retinoic acid (RA) bead (N″), indicating *Crabp2* increase in response to RA. *Crabp2* in quail embryos raised on normal (NQ) (O) or Vitamin A–deficient diet (VADQ) (O′), demonstrating RA-dependence of *Crabp2* transcription in vivo. Tailbud explant pairs (P) cultured in the presence of (P′) control DMSO or (P″) RAR/RXR antagonists shows RA dependence of *Crabp2* expression in late tailbud tissue. Abbreviations as in Figure 1.

As FGF signalling is attenuated by RA provided by somites in the early embryo, we next tested whether the RA synthesising enzyme *Raldh2* continues to be expressed in somites throughout body axis elongation and if expression of RA signalling pathway genes alter in the tailbud. Expression of *Raldh2* was detected in the most recently formed somites throughout body axis elongation (Figure 5A–5D′). However, we additionally observed the appearance of domains of *Raldh2* in the tailbud itself prior to elongation arrest by HH24 (also see [17] for report of whole embryo expression pattern). Tissue localisation of *Raldh2* in sections revealed expression in caudal mesoderm progenitors in the tailbud by HH24 (Figure 5C–5C″). These observations suggest a means by which endogenous retinoid signalling can be locally increased in the tailbud prior to body elongation arrest. Initially transcripts for the retinoic-acid-catabolising enzyme *Cyp26a* were restricted to superficial ventral ectodermal cells, distal notochord, and the CNH at HH16 (Figure 5E–5F′). In contrast, by HH24, *Cyp26a* was lost from the distal notochord/CNH (Figure 5G–5H′), laying this cell population open to an increase in retinoid signalling. *Cyp26a* expression may thus protect the axial stem cells in the CNH from retinoid signalling in the early tailbud.

### Analysis of RA Activity in the Maturing Tailbud

To assess the pattern of retinoid activity, we first analysed the expression of *RARβ* (a canonical Direct Repeat (DR)5 RARE (Retinoic Acid Response Element)–driven gene [43]). However, while *RARβ* was detected in neural tube flanked by somites at all stages, it was only weakly detected in the tailbud at HH24/25 (Figure S2). As *Raldh2* is expressed in the tailbud at this time, these observations support the possibility that the *RARβ* (DR5) RARE does not reveal all sites of retinoid activity [44]. We therefore analysed expression of a gene with different RARE elements, as these might be recognised by distinct combinations of RAR/RXRs [45]. *Cellular retinoic acid binding protein* (*Crabp2*) is an established RA target and its transcription is trans-activated by RARE1 and RARE2 DR motifs that are separated by one (DR1) and two (DR2) base pairs, respectively [43],[45],[46]. The *Crabp2* gene is expressed in early chick and mouse embryos [47]–[50], and here we examined its transcription in the maturing chick tailbud (Figure 5I–5L). From HH15/16, *Crabp2* transcripts are detected at low levels in the tailbud (Figure S3A–S3A′), and detailed analysis from HH19/20 to HH26 revealed that *Crabp2* then steadily encroaches on mesoderm progenitors and the CNH (Figure 5I–5L″″): at HH19–22 *Crabp2* is absent from the CNH but detected in mesoderm progenitors (Figure 5I′–5J″″); by HH24, *Crabp2* is detected in neural/CNH (ventral caudal-most neural tube) and surrounding medial-most mesoderm progenitors, but not the distal-notochord portion of the CNH (Figure 5K′–5K″″); by HH26, a small group of *Crabp2*-expressing cells are then detected in the now abrupt distal end of the notochord (Figure 5L–5L″), while transverse sections through the tail tip at this stage confirmed the multi-lumen nature of the terminating neural tube (Figure 5L′″, 5L″″). These expression patterns correlate well with onset of *Raldh2* and downregulation of *Cyp26a* at HH22–24 and indicate an increase in retinoid signalling in key cell populations of the maturing tailbud (summarized in Figure 5M).

Although *Crabp2* is an established RA target in cultured cells, this has not been demonstrated in the embryo. To test this, RA-delivering beads were implanted between the caudal lateral epiblast/stem zone and presomitic mesoderm in HH9–10 chick embryos (Figure 5N). RA upregulated *Crabp2* expression at the tail-end (*n* = 4/5) compared to control DMSO beads (*n* = 0/5), assessed after 16 h (Figure 5N′–5N″). In addition, tailbuds at HH20/22 treated with 100 nM RA for 24 h in vitro also demonstrated increased *Crabp2* expression in comparison with DMSO-treated controls (Figure S3B–S3B′). To determine whether *Crabp2* transcription depends on RA signalling, *Crabp2* expression was next assessed in Vitamin-A-deficient quail embryos. *Crabp2* transcripts were much reduced in VAD quails (*n* = 3/3) compared to normal quails fixed and processed in parallel (*n* = 7) (Figure 5O–5O′). To specifically test the requirement for RA for *Crabp2* expression in the tailbud, retinoid signalling was blocked using RAR/RXR antagonists; this leads to *Crabp2* repression in 9/12 explant pairs (Figure 5P–5P″). In addition, tailbud explants were treated with the RA synthesis inhibitor disulfiram, and RAR/RXR antagonist delivering beads were transplanted into the HH20/21 tailbud in vivo, and in both conditions *Crabp2* expression was reduced compared with DMSO-treated controls (Figure S3C, S3C′, S3D and S3D′). These data demonstrate that *Crabp2* provides an alternative, D1/D2–RARE-mediated reporter for retinoid signalling in vivo and that it reports increasing RA activity in the tailbud.

### Fgfs Are Repressed by RA and FGF Represses Somitic *Raldh2* During Axis Elongation

**Figure 6:**
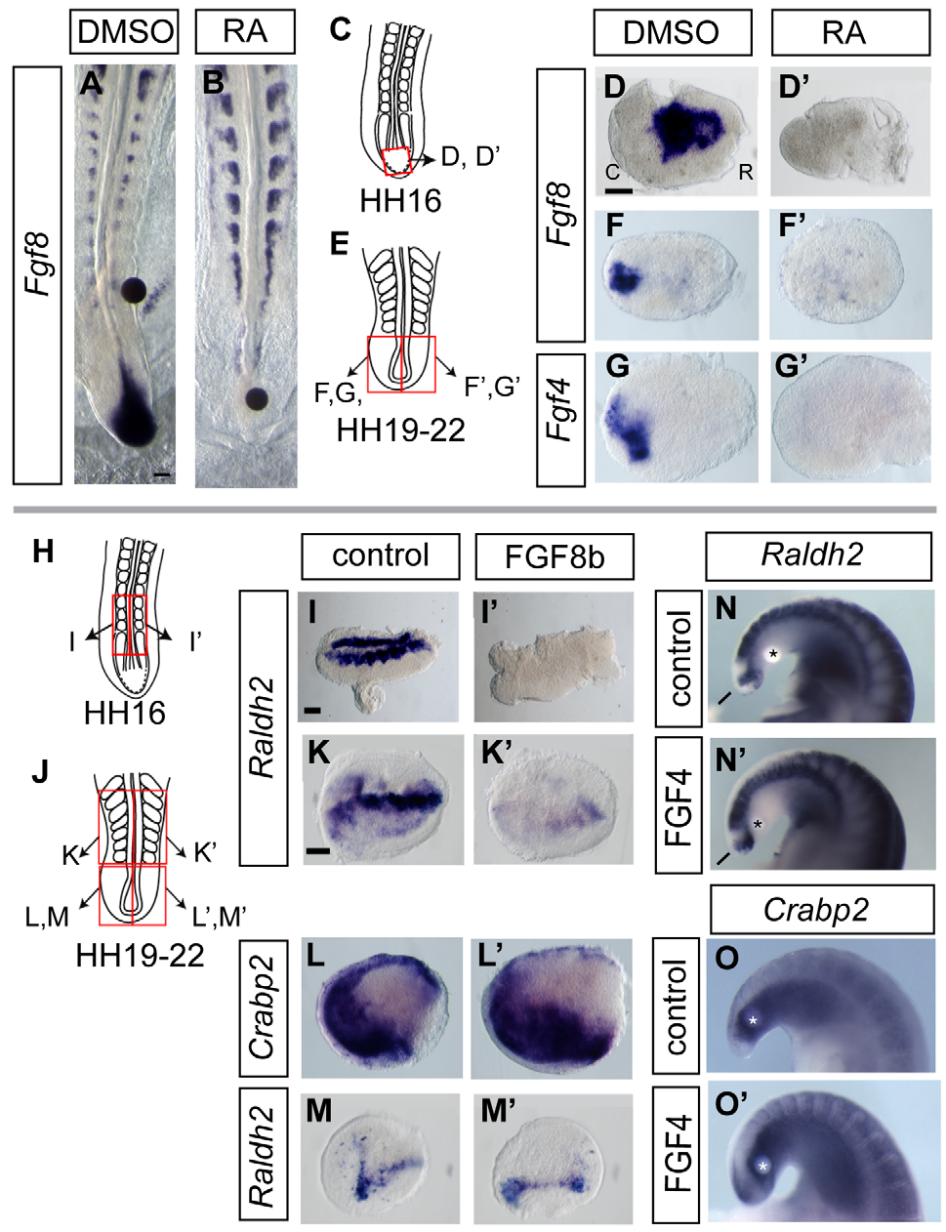
FGF and RA pathway interactions during body axis elongation. Unlike control DMSO beads (A), retinoic acid delivering beads (B) repress *Fgf8* in the early tailbud. Tailbud explanted from HH16 embryos (C) cultured in control DMSO (D) or RA (9-Cis, 10 µM) (D′) and HH19-22 tailbud explants pairs (E) cultured with DMSO (F, G) or RA (1 µM AT-RA, F′; 100 nM AT-RA, G′) showing RA represses *Fgf8* and *Fgf4* in the maturing tailbud. RA inhibited both *Fgf8* and *Fgf4* in these tailbud halves at the lowest concentration of 100 nM AT-RA (*n* = 3/4 explant pairs for each gene). Trunk explant pairs (neural tube flanked by somites and lateral plate mesoderm) from HH16 (H) cultured with BSA vehicle control (I) or FGF8 (I′) lose *Raldh2* in response to FGF8. Older, HH19-22 trunk explant pairs (J) also downregulate *Raldh2* in response to FGF, BSA control (K), and FGF8 treated (K′). HH19–22 tailbud explant pairs (J) do not alter *Crabp2* levels in response to FGF, control BSA (L), and FGF8 (L′). HH19–22 tailbud explant pairs do not alter *Raldh2* levels in response to FGF, control BSA (M), and FGF8 (M′). Explants in (K), (K′), (M), and (M′) are taken from the same embryo, cultured and processed in parallel. In addition, neither BSA nor FGF4 delivering beads repressed expression of *Raldh2* (N, N′) or *Crabp2* (O, O′) expression in the tailbud in vivo. All explants are orientated as in (D). C, Caudal; R, Rostral; asterisks indicate beads.

From the early tailbud (∼HH22) *Crabp2* overlaps with Fgf-responding domains, raising the question of whether FGF and RA signalling maintain their mutually inhibitory interactions in the tailbud. To test the regulatory relationships between these two pathways, we first exposed the prospective tailbud region at HH9–10 to exogenous RA delivered locally on a bead in vivo. This led to loss of *Fgf8* and truncation of the body axis (RA beads *n* = 8/9, DMSO beads *n* = 0/5 embryos) (Figure 6A, 6B). To address the effects of RA on tailbuds at later stages we explanted and cultured tails from HH16 chick embryos for 24 h in DMSO with or without RA. In all cases, while control (DMSO exposed) tissue maintained *Fgf8* expression (*n* = 6), RA-treated tailbuds all lost *Fgf8* transcripts (*n* = 6/6) (Figure 6C–D′). To determine whether RA retains the ability to repress FGF signalling, RA was administered to HH19–22 tailbuds. After 24 h, *Fgf8* and *Fgf4* expression were still detected in control explants (*n* = 9 and *n* = 8, respectively), but in the presence of high (10 µM) or low (100 nM) RA, both these genes were repressed (*Fgf8* 10 µM, *n* = 9/10; *Fgf8* 100 nM, *n* = 3/4; *Fgf4* 10 µM, *n* = 8/8; *Fgf4* 100 nM, *n* = 3/4) (Figure 6E–G′). These data show that retinoid signalling can repress FGF signalling during axis elongation and that it retains this ability in the late stage tailbud.

To test if somitic *Raldh2* can be repressed by FGF signalling at early tailbud stages, pairs of (HH16) trunk explants were dissected from the same embryo and either exposed to BSA vehicle control only or FGF8 (Figure 6H). After 24 h, *Raldh2* is still expressed in somites and adjacent lateral plate in control explants, but in FGF8-treated explants, *Raldh2* is downregulated in both domains (*n* = 8/9 pairs) (Figure 6I–I′). To test this regulatory relationship at later stages, pairs of HH19–22 trunk explants were cultured for 24 h. *Raldh2* was still markedly reduced in all tissues in the presence of FGF8 (*n* = 19/22 pairs) (Figure 6J, 6K, 6K′). Together these experiments indicate that throughout axis elongation caudal FGF signalling continues to repress onset of RA synthesis in forming somites and that *Fgf4* and *Fgf8* transcription in the tailbud remains susceptible to inhibition by RA.

### FGF No Longer Antagonises RA Signalling in the Tailbud

FGF signalling interferes with the RA pathway in the early embryo [2],[12]; however, in the early tailbud, there is an overlap between RA (*Crabp2*) and FGF (*Sprouty2*) activity in mesoderm progenitors. We therefore tested whether FGF retains the ability to block RA activity in the tailbud and found that exposure to Fgf8 protein did not downregulate expression of *Crabp2*, in tailbud pairs (12/12) (Figure 6J, 6L, 6L′). To test whether tailbud *Raldh2* is regulated by FGF, HH19–22 tailbuds were cultured for 24 h with or without FGF8. *Raldh2* was detected in control (*n* = 8/8 pairs) and also FGF8b-treated tailbud explants (*n* = 7/7 pairs) (while control neural tube treated with FGF8b exhibited a reduction in neuron numbers, *n* = 4/4, indicative of active FGF signalling [51]; unpublished data). To confirm that trunk and tail *Raldh2* domains are regulated differently, trunk and tailbud were explanted from the same embryos (Figure 6J, 6K, 6K′, 6M, 6M′). While somitic *Raldh2* was inhibited (*n* = 8/10 pairs), tailbud *Raldh2* was unchanged in all cases (*n* = 10 tailbud pairs). This failure of FGF to repress tailbud *Raldh2* and *Crabp2* transcription was further confirmed by local delivery of FGF4 into the late stage tailbud in vivo (*Raldh2*, 5/5 control BSA beads, 6/7 FGF4 beads; *Crabp2*, BSA beads 5/5, FGF4 beads, 5/5) (Figure 6N, 6N′, 6O, 6O′). In the distinct signalling context of the tailbud, therefore, FGF no longer antagonizes retinoid synthesis or RA activity.

### Retinoid Signalling Attenuates FGF Signalling and Directs Cell Fates in the Tailbud

**Figure 7:**
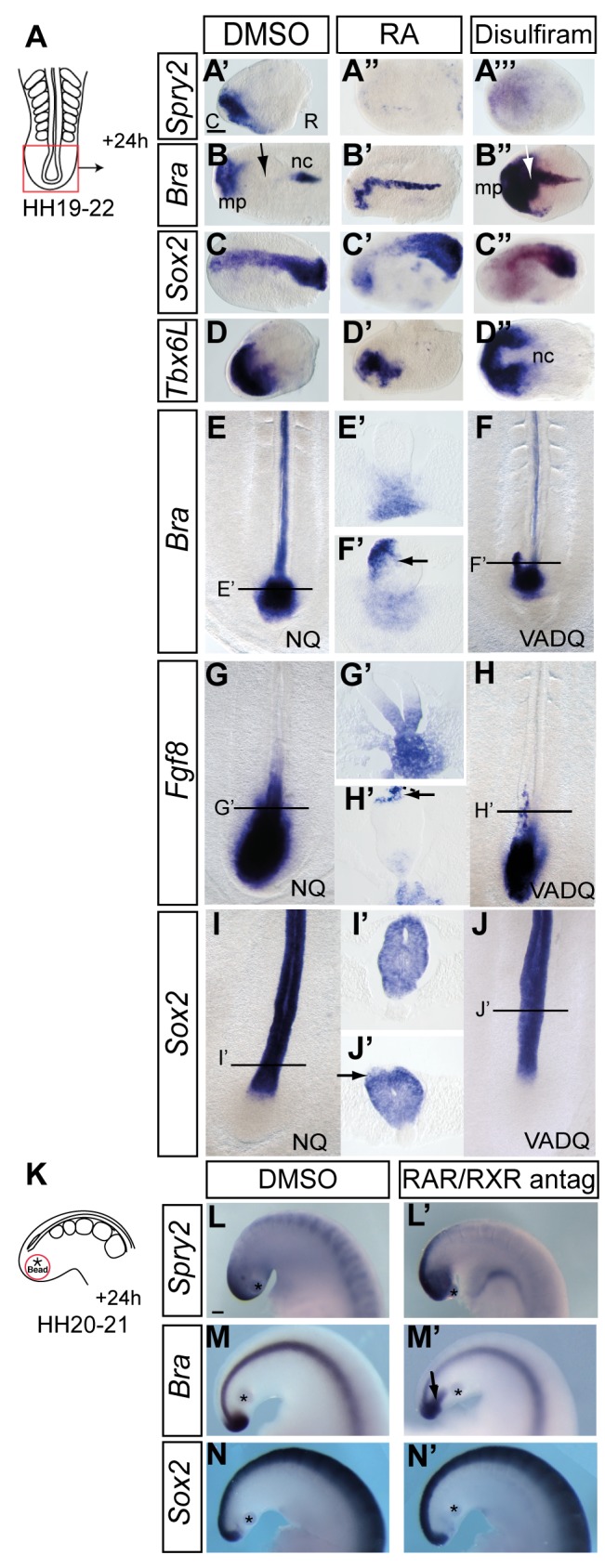
Endogenous retinoid signalling represses mesodermal and promotes neural cell fate in the tailbud. Schematic of whole tailbud explant experiment (A). *Spry2* expression in vehicle control DMSO (A′), retinoic acid (RA) (A″), or retinoic acid synthesis inhibitor Disulfiram (A′″) treated tailbuds. *Bra* expression in DMSO- (B), RA- (B′), or Disulfiram- (B″) treated tailbuds (nc, notochord; mp, mesoderm progenitors; black arrow in B indicates normal downregulation of *Bra* in CNH/medial mesoderm progenitors at ∼HH24 and white arrow in B″ indicates continued expression in these tissues when RA synthesis is inhibited); *Sox2* expression in DMSO- (C), RA- (C′), or Disulfiram- (C″) treated tailbuds. *Tbx6L* expression in DMSO- (D), RA- (D′), or Disulfiram- (D″) treated tailbuds; Quail embryos at HH14–15 were analysed for marker gene expression following development in normal (NQ) and retinoid deficient (VADQ) conditions; mesodermal marker gene *Bra* in normal (E, E′) and retinoid deficient embryos (F, F′), where it is ectopically expressed in the dorsal neural tube (arrow). *Fgf8* in normal (G, G′) and retinoid deficient embryos (H, H′), where it is ectopically expressed in the dorsal neural tube (arrow). Note that the normal quail expressing *Fgf8* is at a slightly later stage and is larger overall than the retinoid deficient quail, however no ectopic patches of *Fgf8* are seen in normal quail neural tube (this section is also not perfectly transverse and is slightly caudal to that shown for in H′; this explains the apparent broader ventral expression of *Fgf8* in the normal quail). Neural progenitor marker *Sox2* in normal (I, I′) and retinoid-deficient embryos, where it is downregulated in dorsal neural tube (arrow) (J, J′). (E′, F′, G′, H′, I′, J′) are transverse sections through the caudal neural tube that contains a lumen. Schematic of in vivo bead implantation experiment (K). *Spry2* expression in the tail bud incubated with vehicle control DMSO (L) or RAR/RXR antagonists beads (L′). *Bra* expression with DMSO (M) or RAR/RXR antagonist (M′) soaked beads. *Sox2* expression with DMSO (N) or RAR/RXR antagonist (N′) soaked beads (*, beads; arrow, ectopic *Bra*).

We have shown that *Fgf4* and *Fgf8* are inhibited in the tailbud by exposure to RA. Consistent with this, we find that treatment of HH19–22 tailbuds with RA (100 nM) for 24 h abolishes expression of *Spry2* and additionally leads to loss of *Bra*, caudal expansion of *Sox2*, and also attenuation of *Tbx6L* in the mesoderm progenitor domain of the tailbud (*Spry2*, DMSO *n* = 0/4, RA *n* = 4/4 [Figure 7A–7A″]; *Bra*, DMSO *n* = 2/11; RA *n* = 8/14 [Figure 7B–7B′]; *Sox2*, DMSO *n* = 2/16, RA *n* = 12/17 [Figure 7C–7C′]; *Tbx6L*, DMSO *n* = 0/7, RA *n* = 7/9 [Figure 7D–7D′]). To test the requirement for endogenous retinoid signalling specifically in the tailbud, we further treated tailbuds with the RA synthesis inhibitor disulfiram, which reduced *Crabp2* expression (Figure S3B, S3B′). This led to expansion of *Spry2* and *Bra* expression in the region of the mesoderm progenitor domain (*Spry2*, DMSO *n* = 0/4, disulfiram *n* = 4/4 [Figure 7A′, 7A″]; *Bra*, DMSO *n* = 0/6, disulfiram *n* = 9/11 [Figure 7B, 7B″]), while *Sox2* expression was reduced caudally (*Sox2*, DMSO *n* = 0/11, disulfiram *n* = 10/13 [Figure 7C, 7C″]), and *Tbx6L* remained strongly expressed, but did not consistently expand its domain in this timeframe (*Tbx6L*, DMSO *n* = 4/4, disulfiram *n* = 4/4 [Figure 7D, 7D″]). These findings suggest that levels of endogenous RA determine FGF activity and subsequent neural versus mesoderm cell fate choice in the mesoderm progenitor domain of the tailbud.

To test whether endogenous RA activity mediates this step in an in vivo context, Vitamin A–deficient (VAD) quail embryos were examined at early tailbud stages HH14–15; retinoid deficiency is embryonic lethal due to heart defects [52],[53], and this was the latest stage at which such embryos were readily available. These embryos exhibit ectopic dorsal domains of *Bra* and *Fgf8* within the neural tube in the caudal-most region (*n* = 0/4 normal quails, *n* = 5/6 VAD quails, *Bra* [Figure 7E–7F′]; and *n* = 0/5 normal quails, *n* = 4/4 VAD quails, *Fgf8*, [Figure 7G–7H′]). *Sox2* expression was detected throughout the neural tube in normal quails (*n* = 4), while asymmetric reduction was observed in the dorsal neural tube of some VAD embryos (*n* = 2/4) (Figure 7I–7J′). These findings indicate that endogenous RA signalling is required for the correct assignment of neural and mesodermal cell fates and for the normal downregulation of *Fgf8* in the early tailbud. To test the continued involvement of retinoid signalling at the latest tailbud stages in vivo, we then grafted beads soaked in RAR/RXR antagonists into the tailbud at HH20–21 and cultured them for a further 24 h (Figure 7K). We first confirmed that delivery of these antagonists in vivo reduced expression of *Crabp2* (Figure S3D, S3D′) (and in more rostral regions *RARb*, DMSO *n* = 0/7, RAR/RXR antagonists *n* = 5/6; unpublished data). We then ascertained that blocking RA signalling in this context lead to increased FGF signalling as indicated by levels of *Spry2* (*Spry2*, DMSO *n* = 1/5; RAR/RXR, *n* = 6/6 [Figure 7L, 7L′]) and to ectopic expression of *Bra* in the tailbud (*Bra*, DMSO *n* = 0/8; RAR/RXR *n* = 3/6 [Figure 7M–7M′]). In addition, *Bra* was also ectopically maintained by exposure to RAR antagonist alone (Figure S4A–S4B′) controlling for potential effects on heterodimers formed by RXRs with receptors unrelated to retinoid signalling. These antagonists did not, however, elicit a consistent change in *Sox2* expression (*Sox2*, DMSO *n* = 6; RAR/RXR *n* = 5 cases [Figure 7N–7N′]), which may reflect maintenance rather than excess levels of FGF signalling in this condition. In conclusion, in all three assays, VAD embryos, tailbuds explanted and treated with disulfiram or exposed to RAR/RXR antagonists in vivo, retinoid deficiency lead to ectopic maintenance of FGF signalling and *Brachyury* expression, supporting a role for endogenous retinoid activity in the loss of FGF signalling and mesodermal cell fate assignment at the end of axis elongation.

### Endogenous Retinoid Signalling Promotes Cell Death Via an FGF-Independent Mechanism

**Figure 8:**
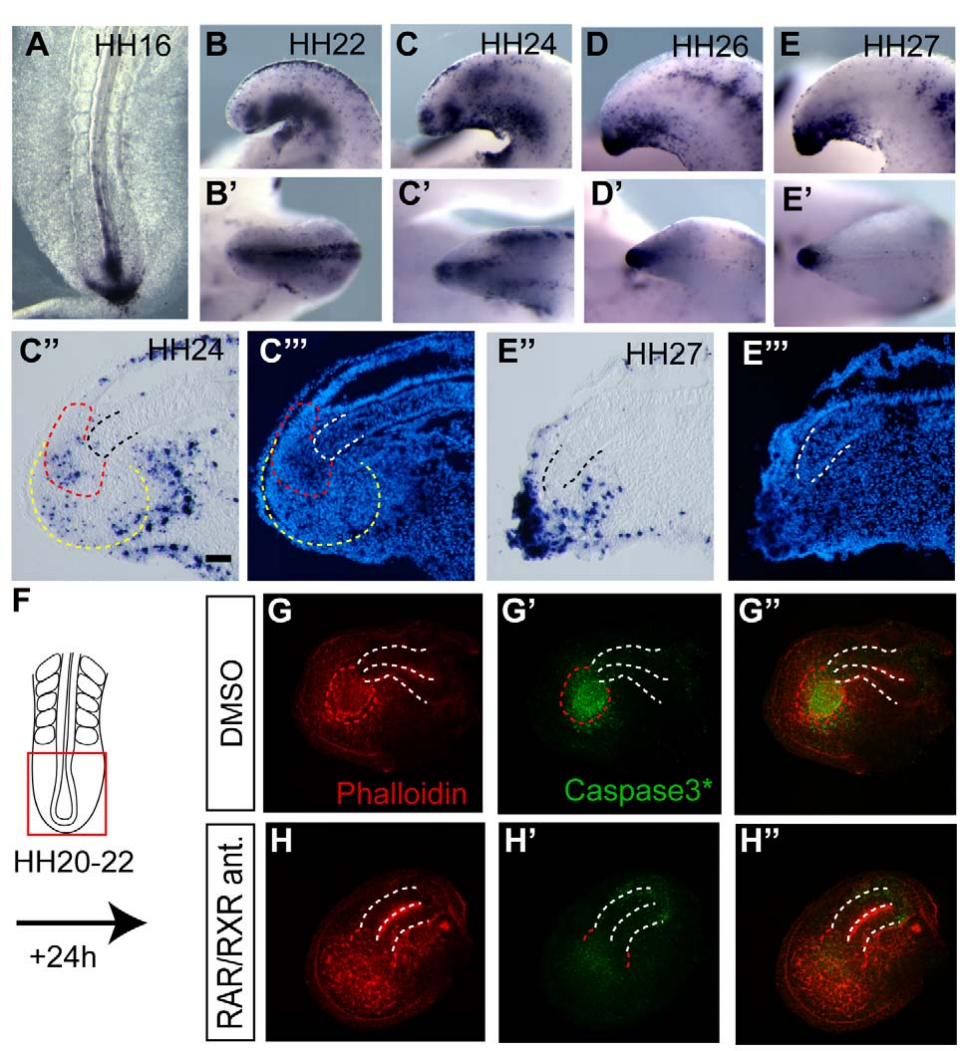
Regulation of cell death in the tailbud by retinoid signalling. (A–E′, C″, E″) Detection of cell death using Tunel (Apoptag) in chick tailbud at key stages (A) HH16, (B, B′) HH22, (C–C′″) HH24, and (D–E′″) HH26/27. (C″) Some apoptotic cells are detected in mesoderm progenitors (yellow dashed line) and the CNH (red dashed line), distal notochord (black dashed line) at HH24. (C′″) section as in (C″) DAPI stained nuclei confirm tissue organisation. (E″) Increase in apoptosis in the terminal structures by HH27, distal notochord (black dashed line). (E′″) section as in E″ DAPI stained nuclei. (F–H″) Cell death detection using NucView 488 caspase-3 substrate (green) and actin cytoskeleton counter-labelling with Phalloidin (red) in HH20 explanted tailbuds cultured for 24 h in (G–G′) control DMSO only conditions or in (H–H″) the presence of RAR/RXR antagonists. White dashed line, neural tube outline; red dashed line, CNH. CNH region is not well defined in RAR/RXR treated tails. This may reflect ectopic/increased Bra in these conditions. Scale bar, 100 µm.

Previous reports show that endogenous cell death increases in the caudal region of the chick embryo during development [15],[54],[55], however this has not been examined with respect to specific tailbud cell populations. The precise timing of apoptosis in individual cells varies within a given tissue, and so TUNEL labelled cells were examined in at least five embryos at each stage to establish the prevailing cell death pattern. Apoptotic cells were detected in the chick-primitive streak from HH10 (unpublished data; and Figure 8A), however only from HH24 in the chick were dying cells present in the CNH and mesoderm progenitors (Figure 8B–8C′″). Dying cells were then detected intensely throughout the tailbud tip by HH26 (Figure 8D–8E′″). This indicates that extensive apoptosis is only active in axial stem and mesoderm progenitor cell populations following increased RA activity after HH24 (compare Figures 5K″ and 8E–8E″).

Previous studies have shown that exposure to excess RA can promote cell death in the tailbud, and this was confirmed by RA treatment of tailbud explants (Figure S5A, S5A′). To investigate whether endogenous retinoid signalling normally regulates cell death, chick tailbuds explanted at HH20–22 were exposed to RAR/RXR inhibitors for 24 h. This revealed a reduction in cell death around and within the CNH (0/8 DMSO; 6/8 RAR/RXR antagonist treated) (Figure 8F–8H″). Although exposure to exogenous FGF8 reduced cell death in explanted tailbud pairs (Figure S6A, S6A′), inhibition of FGFR signalling with PD173074 did not increase cell death in comparison with DMSO controls (Figure S6B, S6B′). These findings show that during normal development apoptosis is only widespread after loss of *Bra* in neural CNH and mesoderm progenitors and *Sox2* expansion in this tissue and suggest that rising endogenous retinoid signalling can promote cell death in the chick tailbud via an FGF-independent mechanism.

## Discussion

This study provides a detailed account of the cellular and molecular changes in the chordo-neural-hinge and mesoderm progenitors in the chick tailbud that underlie the normal process of body axis termination. It reveals a critical loss of mesodermal cell identity in these cell populations in the late tailbud and shows that this is due to a decline in FGFR/Erk signalling, high levels of which can promote a CNH-like cell state. We provide evidence that rising endogenous Retinoid signalling mediates FGF signalling loss and regulates the survival of CNH/axial stem cells and their tailbud derivatives.

Fate maps of the tailbud made with chick/quail chimeras or GFP expressing cells at stage HH15 (25 somites) have shown that medially located cells defined as CNH can still give rise to both neural and mesodermal derivatives [1],[5]. In agreement, we observe that later at HH22 cells located in the equivalent region express both *Bra* and *Sox2*. Moreover, our data reveal a striking loss of the mesodermal gene *Brachyury* specifically in the neural and notochord portions of the CNH and in cells located in the mesoderm progenitor domain. This is accompanied by an expansion of the neural progenitor marker Sox2 into this “mesoderm progenitor” territory in the late (HH24) chick tailbud. Importantly, we show that the neural and notochord CNH persist and remain contiguous with the mesoderm progenitor domain at HH22 (∼44 somite stage) and that cells continue to ingress from the neural CNH into the tailbud at stages leading up to HH24. These findings therefore indicate that the conditions for cessation of body axis elongation arise discretely in the late tailbud and coincide with the end of segmentation at HH24.

This cell state change is coincident with FGF signalling decline in these cell populations, and we demonstrate that blocking FGF/Erk signalling precisely mimics this step, specifically repressing *Bra* in the CNH and mesoderm progenitors and leading to caudal expansion of the *Sox2* expression domain. Consistent with this role for FGF signalling, a recent report analysing the spectrum of phenotypes for human syndromes generated by heterozygous constitutive activation of FGFR2 (Pfeiffer, Crouzon, and Beare-Stevenson syndromes) included an ectopic “caudal appendage” [56], suggesting conservation in humans of a mechanism that attenuates FGFR signalling for normal cessation of body axis elongation. Our finding that the maturing human tailbud also exhibits loss of Bra and terminates in a Sox2-expressing neural structure further supports conservation of the role of FGF in determining cell fates at the end of body axis elongation.

Interestingly, human tails with activated FGFR2 have a variable form, with or without vertebrae [56], indicating that excess FGF signalling can generate ectopic and disorganised tail end structures. This phenotype may depend on the level of FGF activity and relate to our finding of ectopic CNH-like cells co-expressing Bra and Sox2 in response to high-level FGF delivered by beads. Our gain- and loss-of-FGF-function experiments indicate that while loss of FGF promotes neural differentiation, FGF maintains mesoderm progenitors and even higher levels may then promote the axial stem cell state of the CNH. The effects of elevated FGF and Wnt signalling may also explain the large club-end truncation phenotype of Cdx2 mis-expressing mice [57] as Cdx genes are upstream of these pathways [18],[19], which need to be downregulated for segmentation and differentiation in the body axis. Similarly, long-term loss of RA (leading to maintenance of FGF) in the tailbud is not predicted to lead to an ever-lengthening body axis, because RA is also required for subsequent differentiation. Indeed, it is likely that driving FGF only in the CNH would be required for continued axis elongation.

Our findings are consistent with the direct regulation of *Bra* by FGF signalling in lower vertebrates [58], the positive regulatory loop of FGF and Wnt signalling in the early mouse embryo [22] and of Bra and Wnt3a in caudal regions in mouse and zebrafish early embryos [27],[59],[60]. Furthermore, mice mutant for *Bra* exhibit body axis truncation [23],[24], and it may be important that the sudden loss of Bra/Wnt/FGF that we observe coincides with the cessation of segmentation of the presomitic mesoderm in the chick at HH24 [15]–[17]. Indeed, Bra/T box proteins exert direct positive regulation on the Notch pathway ligand *Delta1* and oscillations in this pathway underpin the segmentation process ([61]; reviewed in [62]). The regulation of *Bra* by FGF/Erk signalling thus appears to be a pivotal mechanism that determines vertebrate body length by maintaining mesoderm potential and also by control of a key Notch pathway gene integral to the segmentation of the body axis.

Importantly, using in vivo and in vitro assays, we show that specifically in the tailbud FGF no longer inhibits *Raldh2* expression or RA activity. The downregulation of the p450 enzyme *Cyp26a* in the early tailbud, a known FGF-dependent gene in caudal regions in chick and mouse [13],[26], further suggests that RA is no longer degraded in the chick tailbud. However, we show that RA retains the ability to repress *Fgf4* and *Fgf8* in the tailbud; this could be indirect via loss of Wnt3a, which maintains *Fgf8* (although we also show here that FGF in turn maintains *Wnt3a*, as in the early mouse embryo) [10],[63],[64] and/or directly through binding of an RARE in the *Fgf8* promoter [65],[66]. This breakdown of the oppositional signalling between FGF and RA pathways and the loss of homeostatic regulation of RA turnover provide a mechanism for the steady increase in endogenous RA levels.

We present multiple lines of evidence that rising endogenous retinoid signalling then contributes to loss of FGF/Bra in the tailbud. Using *Crabp2* we show that as FGF signalling declines, endogenous RA activity increases in mesoderm progenitors and the neural CNH and, eventually, in the CNH/notochord distal tip. *Crabp2* transcription is well-established as an RA target in primary cells and cell lines (e.g., human skin fibroblasts [67], F9 teratocarcinoma cells [68], P19 embryonic carcinoma cells [46], and ES cells [69]). It is validated here as a reporter of RA activity in the embryo by its increase in response to exogenous RA delivered by beads or directly on explanted tailbud and its reduction in Vitamin A–deficient quails and inhibition by RA synthesis inhibitor disulfiram and by RAR/RXR antagonists in in vitro and in vivo late stage tailbuds. In these multiple retinoid deficiency assays, we then consistently find ectopic maintenance of FGF signalling and *Bra*, strongly suggesting a role for endogenous retinoid signalling upstream of the loss of mesodermal potential in this tissue. In addition, recent work has confirmed that the chick tailbud is a source of RA, as explanted late-stage tailbuds stimulate expression of an RARb-RARE-LacZ reporter cell line [17],[70]. In the same assay, the mouse tailbud appears not to provide RA, despite also up-regulating *Raldh2* [17],[35]. Most recent data suggest that retinoic acid is not synthesised in the mouse tailbud [35] and so implicate a different mechanism for attenuation of FGF/Wnt signalling as elongation ceases in this animal [4]. This species difference may relate to the retention of the mouse tail, while rising retinoid signalling in the chick additionally regulates cell death and possibly the later normal process of caudal regression in chick and human [71].

Increasing levels of apoptosis were detected in the CNH and medial mesoderm progenitors after HH24. This may serve to remove remaining axial stem and mesoderm progenitor cells but appears too late to be responsible for the localised loss of *Bra* expression. Fate maps of the HH15 tailbud do not indicate that most terminal tissue contributes to neural tube when assessed at HH30 [1], further suggesting that terminal neural tissue identified here may also subsequently undergo apoptosis. A secondary role for cell death in the regulation of axis elongation is consistent with the phenotypes of mice in which RA levels are raised; in *Cyp26a* null embryos, ectopic neural tubes form at the expense of mesoderm, but cell death is not increased in the truncated body axis [29], nor are cell death patterns altered in truncations induced by Cdx mutations [18]. This suggests that endogenous cell death is induced by exposure to higher or longer duration RA and, as inhibition of FGFR signalling did not increase cell death in our assays, may act via a distinct mechanism to promote this further step.

**Figure 9:**
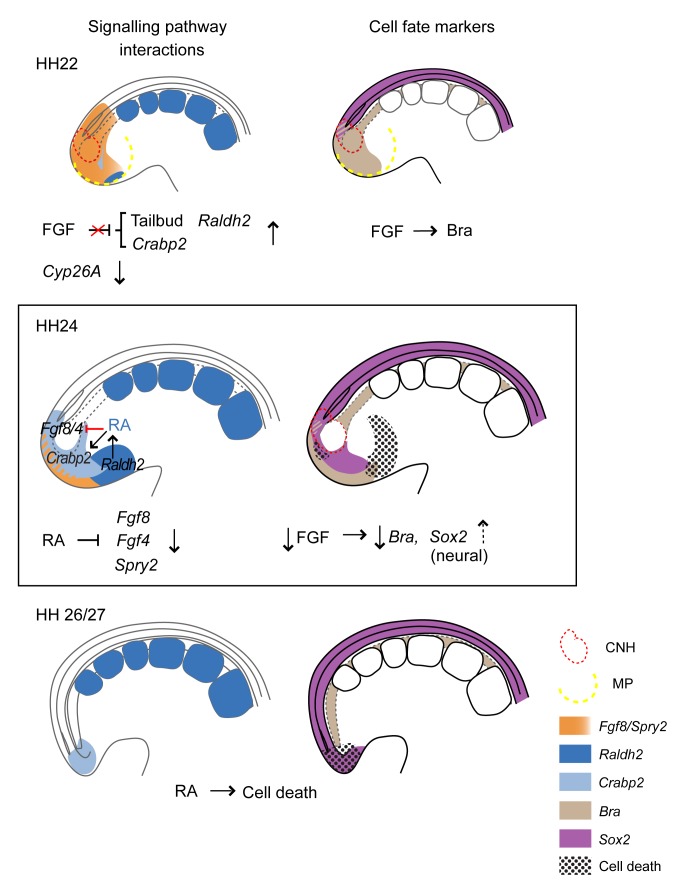
Summary of signalling pathway interactions and cell fate changes underlying body axis elongation arrest. At HH22 *Bra* is expressed in notochord, CNH, and mesodermal progenitors (overlapping with *Sox2* in neural CNH) and is maintained by FGF (and Wnt) signalling, while *Sox2* is confined to neural tube and neural-CNH (see Figure 1A). From this stage *Raldh2* [17] and *Crabp2* begin to be detected in tailbud mesoderm progenitors (signals that induce *Raldh2* here remain to be determined). FGF signalling no longer inhibits *Raldh2* nor represses *Crabp2*, and *Cyp26a* is downregulated; consequently, RA signalling begins to rise in the tailbud. FGF signalling is required to maintain *Bra* (except in proximal notochord) as well as *Tbx6L* and *Wnt3a* (not shown) in mesoderm progenitors. At HH24 FGF signalling suddenly declines, leading to loss of *Bra* and expansion of *Sox2* expression (dashed arrow) into the previous mesoderm progenitor domain, which reflects continued cell ingression from the CNH. At this stage *Crabp2* is now detected in the neural-CNH and position of the mesoderm progenitors, indicating rising retinoid signalling, which can repress FGF ligands and signalling. By HH26/27 FGF signalling is not detected and the notochord ends abruptly encircled by *Sox2* expressing cells. Cell death is widespread in the tailbud and is promoted by retinoid signalling.

In conclusion, these data define a series of regulatory events that control the cessation of body axis elongation in the late stage chick tailbud (Figure 9). It will be important now to identify the signals that promote *Raldh2* expression in the tailbud, what regulatory mechanism blocks retinoid synthesis in mouse tailbud, and the extent to which the mechanisms identified here in the chick are conserved across other vertebrate species. It will further be interesting to assess if the same signalling dynamics are deployed to arrest elongation of other axial outgrowths, such as the limb and beak that are also truncated by excess retinoic acid [72],[73].

## Materials and Methods

### Embryo and Explant Culture and Signalling Pathway Manipulation

Fertile hen's eggs (Henry Stewart) were incubated to required stages after [74]. Early embryos were set up in EC culture [75], and older embryos were accessed in ovo for bead grafts (detailed protocol available on request). AGX-21 beads were soaked in 0.5 mg/ml 9-cis RA or DMSO for controls, and heparin-coated beads were used to present FGF4 (100 µg/ml) or control (bovine serum albumen) BSA. Vitamin A–deficient quails were a kind gift of Malcolm Maden (Kings College London). Explants of chick tissue were isolated as indicated in the text and cultured as described previously [51] with mouse FGF8b (250 ng/ml) (R&D Systems) with heparin (0.1 ng/ml) and BSA (0.0001%); and FGFR inhibitor PD173074 (250 nM) [76] or MEK antagonist PD184352 (3 µM) [77] or DMSO control; 9-cis RA (10 µM or 1 µM, Sigma) or all-trans RA (10 µM, 1 µM, or 100 nM, Sigma) had similar effects and were used as indicated in figure legends or control DMSO, RA synthesis inhibitor Disulfiram (1 or 10 µM), or RAR and RXR antagonists LG100815 and LG101208 (5 µM) (Ligand Pharmaceuticals), or DMSO control, as described previously [11].

### Implanting Beads into Stage Late Stage Tail Bud in Vivo

HH stage 20/21 embryos were accessed in ovo for bead grafts. AGX-21 beads were soaked in RAR and RXR antagonists LG100815 and LG101208 (1 mM) (Ligand Pharmaceuticals) or DMSO for controls, and heparin-coated beads were used to present FGF4 (100 µM) or control BSA. After opening the egg shell, the vitelline membrane, blood vessels, and the amnion around the tail bud region were gently displaced/peeled away with fine forceps. The tail bud was lifted and stabilised on its side by placing a thin slice of agarose gel (2% agarose, 1×PBS, 0.01% Fast green) beneath it (see Figure 4F). A small opening was made in the caudal tail bud using a tungsten needle into which a bead was implanted. The agar was then slid away and the tailbud eased back into its normal vertically hanging position, the egg sealed, and re-incubated for 24 h.

### Fate Mapping

DiI labelling of cell groups was carried out as in [78]. DiI (1,1′-dioctadecyl-3,3,3′,3′ tetram-ethyl-indocarbocyanineperchlorate, Cell Tracker CM-DiI, Invitrogen) 1 µg/µl in DMF (N,N-dimethylformamide) was used to label small cell groups in the caudal most neural tube, the chordo-neural-hinge, and mesoderm progenitors in the stage 20/21 embryos. DiI was injected by glass capillary after temporary stabilization of the exposed tail bud on the agarose gel as described above. Following labelling, cell position was confirmed, agar removed, and tailbud eased back into position before sealing the egg and incubation for a further ∼30 h (a subset of embryos were fixed immediately, imaged, and sectioned to determine labelling accuracy; see Figure 3 legend). Embryos were then fixed in 4% PFA for 2 h and washed in PBS. Whole tails were imaged before sectioning and analysis (see Table 1).

### In Situ Hybridisation and Immunocytochemistry

Standard methods for whole mount in situ hybridisation were used. Explanted tissues were processed using an InsituPro machine (Intavis). Normal expression patterns were observed in at least four embryos per stage, per gene. Tailbud cell populations were analysed in sections prepared following standard cryosectioning. Immunocytochemistry in human embryos at CS12-17 for Sox2 and Bra was carried out using a standard protocol for wax sections, using citrate buffer antigen retrieval. Primary antibodies were as follows: Goat anti-Sox2 Immune Systems Ltd (GT15098; batch 401196) (1∶100); Rabbit anti-Brachyury (Santa Cruz) (SC-20109) (1∶50); and for Chick sections, Rabbit anti-Sox2 (Millipore Ab 5603) (1∶100) and Goat anti-Brachyury R&D Systems. Secondaries were all from Molecular Probes and used at 1∶200; for Human tissue, Donkey anti-Goat A594 and Donkey anti-rabbit-A488; and for Chick tissue, Donkey anti-Goat A488 and Donkey anti-Rabbit A594. Labelled sections were imaged using a Delta-Vision widefield microscope.

### Cell Death Assay

The ApopTag In Situ Apoptosis Detection Kit (Chemicon) or NucViewTM 488 Caspase-3 substrate, a novel, fixable cell membrane-permeable fluorogenic caspase substrate that detects Caspase-3 activity within live cells (Biotium, 30029), was used according to the manufacturers' instructions.

### Cloning

A chick *Crabp2* probe was generated from ChEST74f2 (BBSRC Chicken EST database). ChEST74f2 was sequenced and coding region (414 bp) and short flanking sequences identified. Blast searches indicated a single match to crabp2-like gene in meleagris gallopavo (accession number XM 003213787.1).

## Supporting Information

Figure S1 *Sox2* expression at HH20–21. *Sox2* transcripts are detected transiently in the chick distal tail gut and are lost at HH20/21, after which this structure disappears and *Sox2* is only detected in the neuroepithelium and at HH24 spreads into the position of the mesoderm progenitor domain (see Figure 4).
(TIF)

Figure S2 Expression of *RARβ* in the chick tailbud. In situ hybridisation for *RARβ* from middle to end of body axis elongation in chick (A–D′). Low-level *RARβ* is detected in the chick tailbud. In all figures, stages are labelled; top rows are lateral views, bottom rows dorsal views. (A′) and (C″) are sagittal sections at the indicated stages. CNH, red dashed line; mesoderm progenitors, yellow dashed line; nt, neural tube; nc, notochord. Scale bars are 100 µm.
(TIF)

Figure S3 *Crabp2* expression and regulation by retinoid signalling in the tailbud. *Crabp2* expression at HH10 (A) and HH15/16 (A′) is not detected in the tailbud. HH19–22 tailbud explants treated with DMSO-only control (B) or RA (all trans RA, 100 nM) (B′) in which *Crabp2* expression is increased (*n* = 2/2). HH19–22 tailbud explants treated with (C) DMSO only or the RA-synthesis inhibitor Disulfiram (C′) in which *Crabp2* expression is reduced (*n* = 5/5). Beads delivering control DMSO (D) or RAR/RXR antagonists (D′) were grafted into the HH20/21 tailbud and cultured for 24 h. RAR/RXR antagonists reduced *Crabp2* expression (*n* = 6/8), compared to DMSO controls (1/6). RAR antagonist beads placed in the flank attenuate RARb and Crabp2 expression. Scale bars, 100 µm. *, bead position.
(TIF)

Figure S4 RAR antagonist alone elicits ectopic *Bra* expression. Schematic of bead grafting experiment (A), DMSO (B), and RAR antagonist delivering beads (B′). Ectopic *Bra* was detected in response to RAR antagonist (*n* = 5/12) but not DMSO beads (*n* = 0/8). Scale bar, 100 µm. *, bead position.
(TIF)

Figure S5 RA increases cell death in tailbud explants. (A) HH19–22 tailbud explant pairs were cultured either in DMSO (B) alone or in RA (B′) and exhibited increased apoptosis as indicated by Caspase activity using NucView TM 488, a fluorogenic Caspase substrate based assay, in the presence of 10 µM RA (*n* = 3/3 explant pairs). At lower RA concentrations, the increase in cell death was less pronounced; 3/5 explant pairs cultured in 1 µM atRA and 2/5 pairs at 0.1 µM atRA (unpublished data).
(TIF)

Figure S6 FGF regulation of apoptosis in the tailbud. (A, A′) Exposure of HH19–22 tailbud explants to FGF8b (200 ng/ml) for 24 h reduced incidence of cell death as indicated by Caspase-3 activity (NucView) in comparison with no growth factor control conditions (*n* = 4/4 explant pairs). (B, B′) Exposure of HH19–22 tailbud explants to FGFR inhibitor PD173074 did not consistently alter levels of apoptosis as indicated by Caspase-3 activity, in comparison with control DMSO-only-treated tailbuds (PD173074, *n* = 6; DMSO, *n* = 4). Explants for FGF8 were imaged on a conventional wide-field microscope, and PD173074 experiments were imaged using confocal microscopy.
(TIF)

## Abbreviations

Bra: Brachyury
CNH: chordo-neural-hinge
Crabp2: Cellular retinoic acid-binding protein 2
CS: Carnegie Stage
Cyp26A: Cytochrome P450 26A1
DiI 1: 1′-dioctadecyl-3,3,3′,3′ tetram- ethyl-indocarbocyanineperchlorate
DMSO: Dimethyl sulfoxide
DR: direct repeat
FGF(R): Fibroblast growth factor (receptor)
HH: Hamburger and Hamilton
MEK: Mitogen-activated protein kinase kinase
RA(R): retinoic acid (receptor)
Raldh2: retinaldehyde dehydrogenase 2
RARE: retinoic acid response element
Sox2 SRY (sex determining region Y) box 2
Spry2: Sprouty2
Tbx6L: T-box-containing protein 6L
VAD(Q): vitamin A deficient (quail)
