# Supplementary figures and images for: Loss of FGF-Dependent Mesoderm Identity and Rise of Endogenous Retinoid Signalling Determine Cessation of Body Axis Elongation
Source: PLoS Biol. 2012 Oct 30;10(10):e1001415. doi: 10.1371/journal.pbio.1001415 (PMC3484059; doi:10.1371/journal.pbio.1001415)

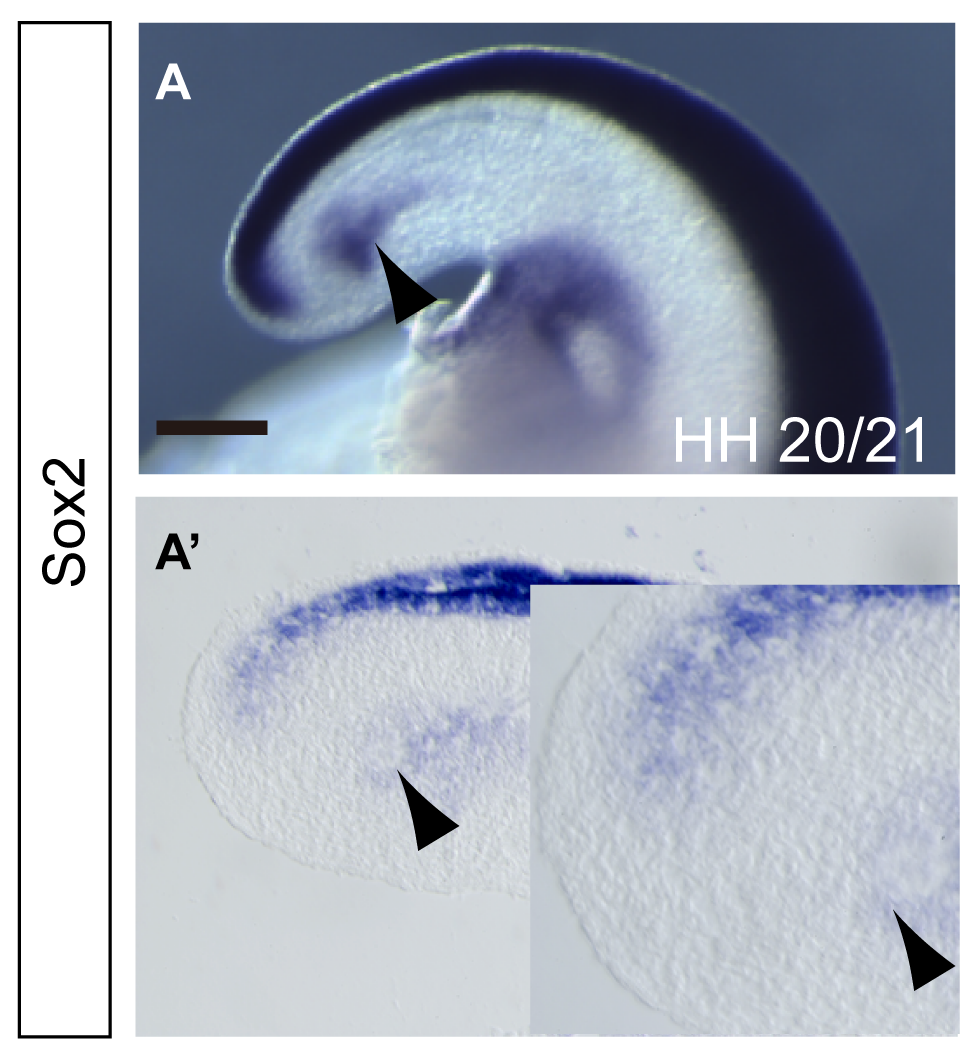

Supplement: Figure S1 — Sox2 expression at HH20–21. Sox2 transcripts are detected transiently in the chick distal tail gut and are lost at HH20/21, after which this structure disappears and Sox2 is only detected in the neuroepithelium and at HH24 spreads into the position of the mesoderm progenitor domain (see Figure 4). (TIF) [file pbio.1001415.s001.tif]

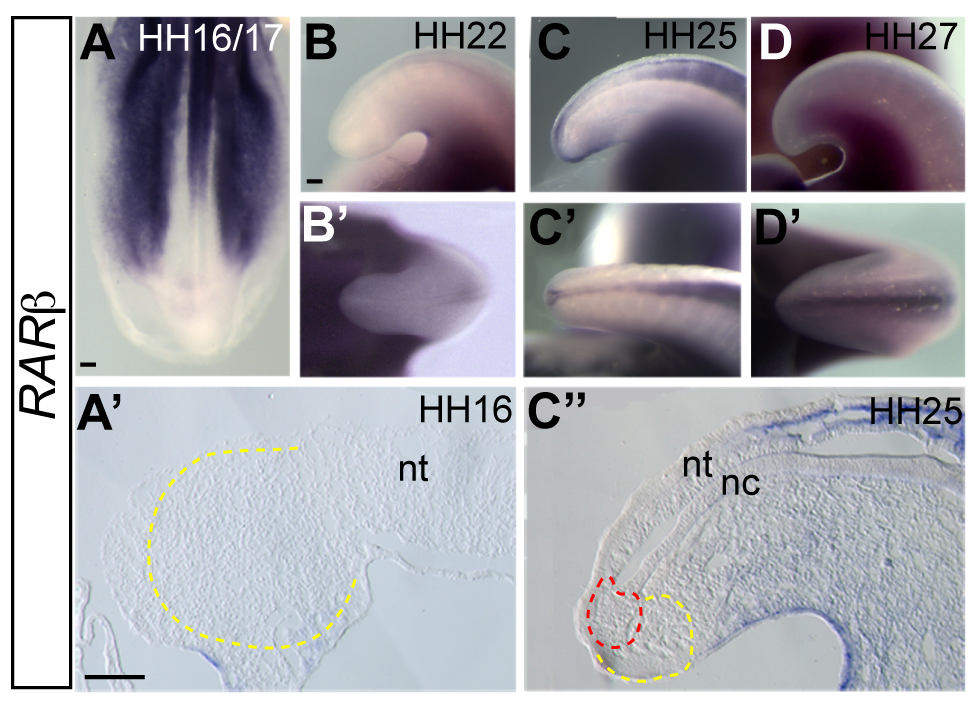

Supplement: Figure S2 — Expression of RARβ in the chick tailbud. In situ hybridisation for RARβ from middle to end of body axis elongation in chick (A–D′). Low-level RARβ is detected in the chick tailbud. In all figures, stages are labelled; top rows are lateral views, bottom rows dorsal views. (A′) and (C″) are sagittal sections at the indicated stages. CNH, red dashed line; mesoderm progenitors, yellow dashed line; nt, neural tube; nc, notochord. Scale bars are 100 µm. (TIF) [file pbio.1001415.s002.tif]

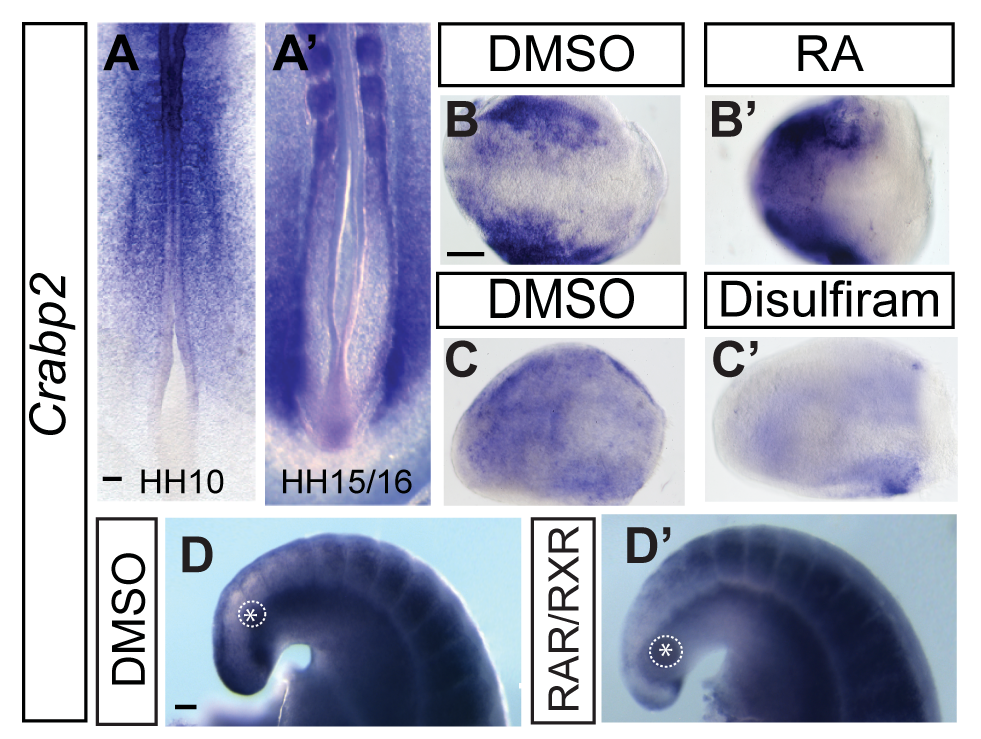

Supplement: Figure S3 — Crabp2 expression and regulation by retinoid signalling in the tailbud. Crabp2 expression at HH10 (A) and HH15/16 (A′) is not detected in the tailbud. HH19–22 tailbud explants treated with DMSO-only control (B) or RA (all trans RA, 100 nM) (B′) in which Crabp2 expression is increased (n = 2/2). HH19–22 tailbud explants treated with (C) DMSO only or the RA-synthesis inhibitor Disulfiram (C′) in which Crabp2 expression is reduced (n = 5/5). Beads delivering control DMSO (D) or RAR/RXR antagonists (D′) were grafted into the HH20/21 tailbud and cultured for 24 h. RAR/RXR antagonists reduced Crabp2 expression (n = 6/8), compared to DMSO controls (1/6). RAR antagonist beads placed in the flank attenuate RARb and Crabp2 expression. Scale bars, 100 µm. *, bead position. (TIF) [file pbio.1001415.s003.tif]

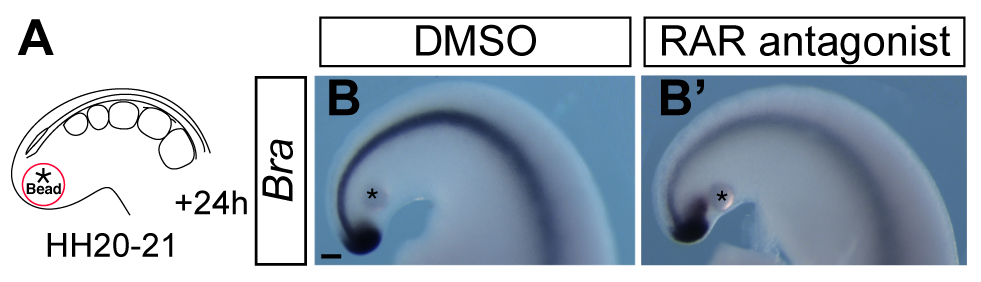

Supplement: Figure S4 — RAR antagonist alone elicits ectopic Bra expression. Schematic of bead grafting experiment (A), DMSO (B), and RAR antagonist delivering beads (B′). Ectopic Bra was detected in response to RAR antagonist (n = 5/12) but not DMSO beads (n = 0/8). Scale bar, 100 µm. *, bead position. (TIF) [file pbio.1001415.s004.tif]

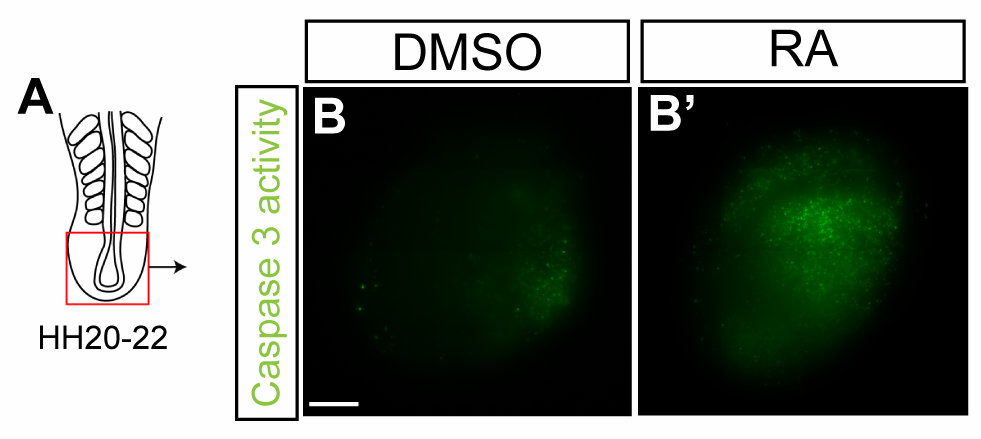

Supplement: Figure S5 — RA increases cell death in tailbud explants. (A) HH19–22 tailbud explant pairs were cultured either in DMSO (B) alone or in RA (B′) and exhibited increased apoptosis as indicated by Caspase activity using NucView TM 488, a fluorogenic Caspase substrate based assay, in the presence of 10 µM RA (n = 3/3 explant pairs). At lower RA concentrations, the increase in cell death was less pronounced; 3/5 explant pairs cultured in 1 µM atRA and 2/5 pairs at 0.1 µM atRA (unpublished data). (TIF) [file pbio.1001415.s005.tif]

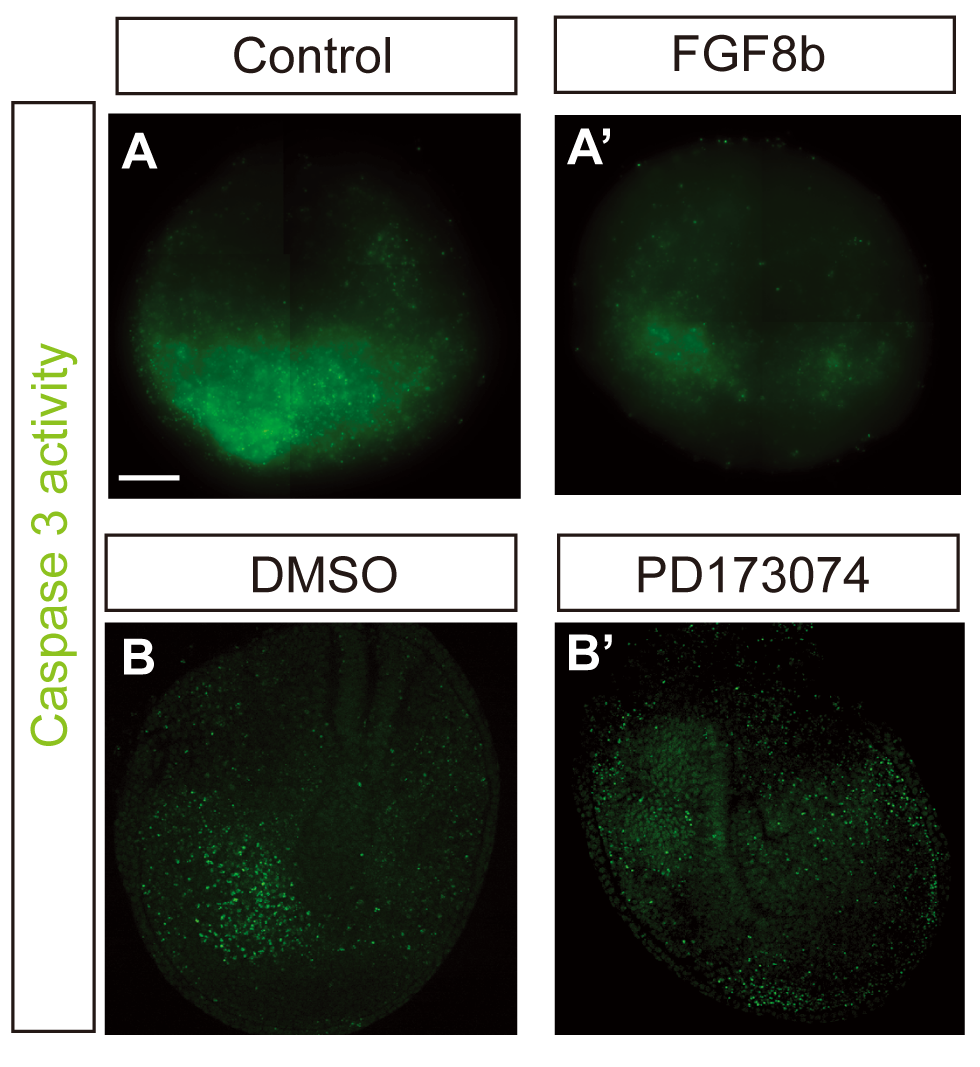

Supplement: Figure S6 — FGF regulation of apoptosis in the tailbud. (A, A′) Exposure of HH19–22 tailbud explants to FGF8b (200 ng/ml) for 24 h reduced incidence of cell death as indicated by Caspase-3 activity (NucView) in comparison with no growth factor control conditions (n = 4/4 explant pairs). (B, B′) Exposure of HH19–22 tailbud explants to FGFR inhibitor PD173074 did not consistently alter levels of apoptosis as indicated by Caspase-3 activity, in comparison with control DMSO-only-treated tailbuds (PD173074, n = 6; DMSO, n = 4). Explants for FGF8 were imaged on a conventional wide-field microscope, and PD173074 experiments were imaged using confocal microscopy. (TIF) [file pbio.1001415.s006.tif]
